# Tuning recombinant protein expression to match secretion capacity

**DOI:** 10.1186/s12934-018-1047-z

**Published:** 2018-12-22

**Authors:** Luminita Gabriela Horga, Samantha Halliwell, Tania Selas Castiñeiras, Chris Wyre, Cristina F. R. O. Matos, Daniela S. Yovcheva, Ross Kent, Rosa Morra, Steven G. Williams, Daniel C. Smith, Neil Dixon

**Affiliations:** 1 Manchester Institute of Biotechnology, School of Chemistry, University of Manchester, Manchester, M1 7DN UK; 2 Cobra Biologics Ltd, Keele, ST5 5SP UK

**Keywords:** Periplasmic secretion, Antibody fragments, Riboswitches, Codon usage, Signal peptides

## Abstract

**Background:**

The secretion of recombinant disulfide-bond containing proteins into the periplasm of Gram-negative bacterial hosts, such as *E. coli,* has many advantages that can facilitate product isolation, quality and activity. However, the secretion machinery of *E. coli* has a limited capacity and can become overloaded, leading to cytoplasmic retention of product; which can negatively impact cell viability and biomass accumulation. Fine control over recombinant gene expression offers the potential to avoid this overload by matching expression levels to the host secretion capacity.

**Results:**

Here we report the application of the *RiboTite* gene expression control system to achieve this by finely controlling cellular expression levels. The level of control afforded by this system allows cell viability to be maintained, permitting production of high-quality, active product with enhanced volumetric titres.

**Conclusions:**

The methods and systems reported expand the tools available for the production of disulfide-bond containing proteins, including antibody fragments, in bacterial hosts.

**Electronic supplementary material:**

The online version of this article (10.1186/s12934-018-1047-z) contains supplementary material, which is available to authorized users.

## Background

Microbial cells have evolved phenotypic traits and cellular functions matched to their endogenous environmental niches; however they have not necessarily evolved with the cellular production capacity requirements often demanded in a biotechnological context. With respect to recombinant protein production, host cells are required to produce large quantities of heterologous protein, but may not exhibit the appropriate intracellular processing capacity to match this biotechnological demand imposed upon them. For example, they may not exhibit the required cellular synthetic capacity, folding capacity or indeed secretion capacity. In such scenarios high levels of recombinant protein production overload the host’s capacity resulting in deleterious outcomes for the recombinant protein and/or the production host [1–7]. A number of potential solutions are available to address these imbalances: (i) increase the host’s capacity, e.g. by overexpression of endogenous genes encoding helpers proteins such as chaperones, secretion machinery, and ancillary factors, (ii) add new capability e.g. expression of heterologous genes encoding helper proteins, or (iii) seek to match expression demand with the host’s capacity [3, 8–11].

Secretion of recombinant protein offers a number of potential advantages. By allowing segregation of the protein product away from the cytoplasmic components to (i) reduce the chance of any deleterious interactions of the recombinant protein with the host and reduce molecular crowding effects, (ii) reduce the exposure of the recombinant protein to host cytoplasmic proteases, (iii) aid disulfide bond formation, away from reducing cytoplasmic environment, and (iv) produce recombinant proteins with a true N-terminus (absence of methionine). In Gram-negative bacteria protein secretion across the inner membrane into the periplasmic space occurs predominantly via the SecYEG translocon [12]. Pre-proteins containing a N-terminal signal sequence (signal peptides), of 18–30 amino acids in length, target the proteins for secretion [13]. The hydrophobicity of the signal peptide determines whether secretion occurs via the SecB-dependent or the signal recognition particle-dependent (SRP) pathway [14]. The classical distinction is that translocation via the SecB path occurs post translation, and the SRP path via co-translation. Both the SecB and SRP pathways maintain the pre-protein in an unfolded ‘translocation-competent state’ [15]. Both pathways involve 3 key steps, (i) sorting and targeting, (ii) translocation, and (iii) release. The efficiency of each step is dependent upon the dynamic, transient interactions between the target protein and the various stages of the respective pathways, and hence secretion efficiency is highly dependent upon the biophysical characteristics of the recombinant protein [12, 14, 15].

Although Sec-dependent secretion is widely used, there are well-documented examples where the secretion machinery becomes overloaded and the Sec translocon becomes ‘jammed’ resulting in accumulation of the target protein in the cytoplasm and cell toxicity [16]. Above a certain optimal rate of translation, secretion rates can rapidly decrease [17]. This is most likely due to the limited secretion capacity of the *E. coli* transport machinery compared to the rate of translation [5]. When this secretion capacity is overwhelmed, the excess target protein is likely to accumulate in inclusion bodies, affecting protein titres and cell viability, highlighting the need to carefully optimize expression levels and rate of recombinant protein production [18].

**Fig. 1:**
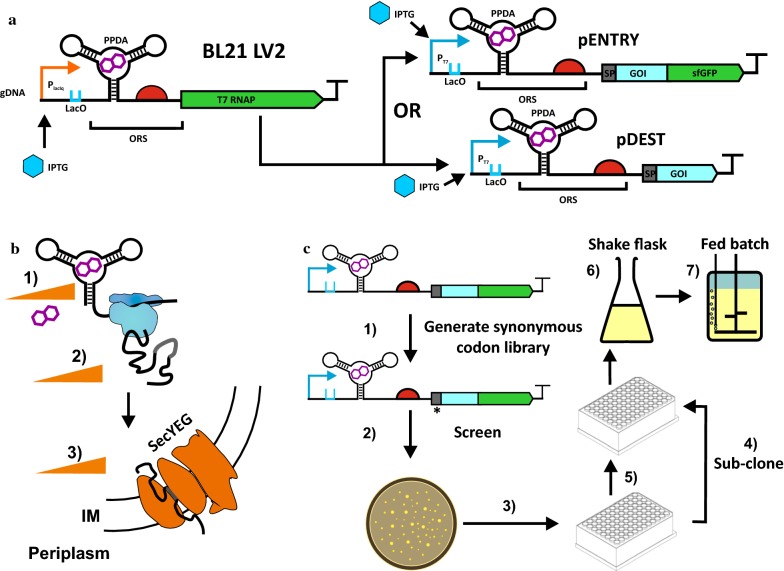
Concept and workflow of applying the *RiboTite* expression system for titratable secretion. **a** The *RiboTite* system operates at both the transcription and translation level, to afford a gene regulatory cascade controlling both T7 RNAP and the gene of interest (GOI). Transcriptional control is mediated by the lacI repressor protein, induced by Isopropyl β-D-1-thiogalactopyranoside (IPTG). Translational control is mediated by an orthogonal riboswitch (ORS) which releases and sequesters the ribsome binding site (RBS) in the presence and absence of the inducer Pyrimido-pyrimidine‐2,4‐diamine (PPDA) respectively. The system is composed on an *E. coli* expression strain BL21(LV2) strain, and expression plasmids containing the T7 promoter. Shown are the pENTRY and pDEST expression plasmids used that incorporate the signal peptide sequence (SP) to direct the produced protein for periplasmic translocation, and GOI and GOI-sfGFP fusions also under orthogonal riboswitch (ORS) and T7 promoter control. For further description of the BL21(LV2) cassette see Additional file 1: Fig. S1. **b** Riboswitch-dependent translation control of the *RiboTite* system is employed to match expression rate to the secretion capacity of the Sec pathway. **c** Schematic diagram of workflow. The pENTRY vectors were used to integrate the 5′UTR riboswitch with the 5′ encoded SP sequences. (1) A synonymous codon signal peptide library was generated, and (2–3) screened to select for clones that exhibit high protein expression and high regulatory control over basal induction. (4) Selected clones were sub-cloned into the pDEST vectors, and (5) screened for expression and secretion at small scale in shaker flasks (6) and in fed-batch bioreactors (7)

The commonly employed inducible bacterial expression systems mostly operate at the transcriptional level. For instance, lactose or arabinose regulated systems generate a heterogeneous cell population upon induction, where some cells are fully induced and other cells remain un-induced [19, 20]. Tuneable expression systems can address some of these limitations by modulation of gene expression to adjust to the physiological needs of the bacterial host and provide optimal parameters for recombinant protein production [21, 22]. The *RiboTite* technology has been demonstrated to robustly control the expression of a variety of recombinant genes encoding therapeutic proteins in *E. coli,* and provides cellular level titratable control of gene expression and very tight control of basal gene expression in the absence of induction [23]. The system operates at both the transcription and translation level to afford a gene regulatory cascade, by using an inducible promoter-operator-repressor (IPTG, P/O_lac_, lacI), and a small molecule Pyrimido-pyrimidine‐2,4‐diamine (PPDA) inducible translational ON orthogonal riboswitch (ORS), to control both a chromosomal copy of T7 RNAP and an episomal copy of recombinant gene of interest (GOI) (Fig. 1a).

Production of therapeutically important proteins such as cytokines and antibody fragments in *E. coli* commonly employs the SecYEG translocon to secrete the proteins into the periplasmic space [24, 25]. Antibody fragments are truncated and engineered versions of antibodies, usually derived from the IgG isotype, contain the complementarity-determining regions (CDRs) that retain binding capacity to specific antigens [26]. A single chain Fv (scFv) consists of heavy and light chain association through a short synthetic peptide linker. Antibody fragments have extensive applications for diagnostics and detection of a wide repertoire of agents, as well as for therapeutic treatment of a range of health disorders [27]. A range of scFv agents and derivatives are currently in clinical trials, with one anti-VEGF scFv that successfully completed Phase III trials in 2017 [28, 29]. In this study, we explored whether the precise control of gene expression offered by the *RiboTite* system would avoid the previously observed overload of the Sec translocon [5, 16], and permit isolation of protein with increased product quality, activity and titres.

## Results

### Concept and workflow of applying the *RiboTite* expression system for titratable secretion

The *RiboTite* expression system [23] was employed in order to regulate SecYEG-dependent secretion of single chain antibody fragments (scFv) into the periplasm of *E. coli* (Fig. 1b). Here expression plasmids were constructed where the gene of interest (GOI) was placed in-frame with sfGFP to generate a fusion protein (pENTRY) (Fig. 1c). The pENTRY permits rapid evaluation and selection of signal sequence variants from a synonymous codon library. Following selection of variants with enhanced expression and regulatory performance, the fusion protein was removed by sub-cloning the GOI into pDEST plasmid and secretion performance was assessed. The selected clones were then assessed under fed-batch fermentation control to validate their performance under high cell density culture conditions. In this study we utilised the single chain antibody fragments anti-β-galactosidase (scFvβ) [30], anti-histone (scFvH) [31], and anti-tetanus (scFvT) [32].

### Design and construction of the expression strain and plasmids

In this study expression strains and plasmids were developed to simultaneously achieve enhanced basal control and integration of the 5′ encoded signal peptide sequence respectively. The *E. coli* expression strain BL21(LV2), was designed from the previously reported BL21(IL3) strain [23], by (i) replacing the repressor gene with a stronger repressor (*lacI*^*q*^), (ii) inverting its orientation to the opposite direction to the T7 RNAP gene, and (iii) incorporating an additional operator (O3) to further tighten the basal expression (Additional file 1: Fig. S1). To assess this modification we benchmarked performance of various T7 RNAP-dependent strains for expression and regulatory control (Additional file 1: Table S1, Fig. S2). The analysis was performed by monitoring expression of eGFP (cytoplasmic) under different induction conditions, times and growth media. The BL21(LV2) strain demonstrated total expression comparable to the most commonly used expression strain BL21(DE3), but with significantly greater regulatory control (> 1000-fold vs. ~ 30-fold) in the presence of the respective inducers, and was used for all subsequent analysis.

Expression-secretion plasmids (pENTRY, pDEST) were designed to direct the produced recombinant protein towards the SecYEG translocon for periplasmic secretion. Four different signal peptide encoding sequences (SP) were cloned upstream of the GOI: two SecB-dependent signal peptides (Piii and PelB) and two SRP-dependent signal peptides (DsbA and yBGL2) [33, 34].

### Integration of signal peptide sequences with the regulatory *RiboTite* system permits tuneable control of gene expression

The performance of *cis*-encoded regulatory RNA devices is known to be highly sensitive to flanking nucleotide sequence and structure [35, 36]. This poor modulatory limits the facile integration of RNA devices, e.g. riboswitches into alternative coding contexts. Close to an open reading frame RNA regulatory performance e.g. translation initiation from the ribosome-binding site (RBS) has been shown to be sensitive to secondary structure in the 5′ coding region [37–39]. Building on this approach we recently developed a riboswitch integration method that permits selection of codon variants with expanded riboswitch-dependent regulatory control over gene expression [40]. To optimise the regulatory performance of the *cis*-encoded translation ON riboswitch located in the 5′UTR and 5′ encoded signal peptide sequences, the recently developed codon context integration method was used [40]. The method is based on the introduction of synonymous codons immediately downstream from the start codon; this conserves the amino acid sequence of the resulting signal peptide that interacts with the secretory apparatus (i.e. SRP or SecB), whilst permitting codon usage and RNA folding space to be explored.

**Fig. 2:**
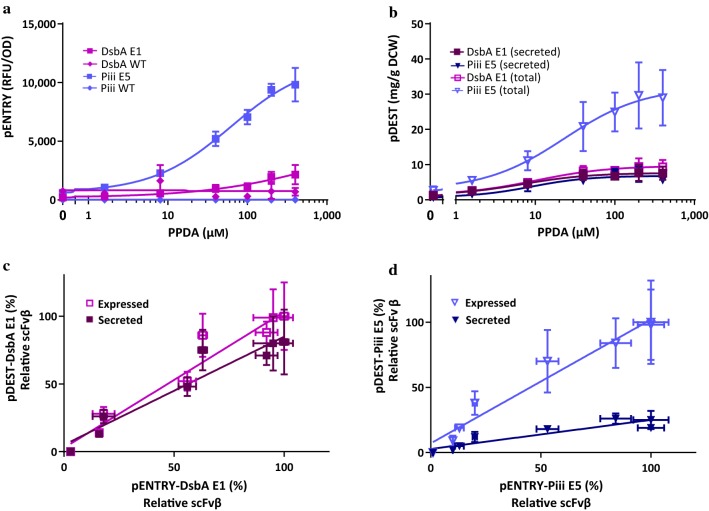
scFvβ expresion control and correlation analysis for codon-optimised signal peptide sequences constructs. **a** Dose response curves for pENTRY constructs, relative fluorescent units normalized to cell density (RFU/OD_600_) against inducer concentration for the WT and codon optimised DsbA and Piii signal peptide sequences. **b** Dose response curves for pDEST constructs, expression yield per cell reported as mg per g dry cell weight. **c** Linear regression of the pENTRY expression vs. pDEST expression for DsbA-E1 and Piii-E5. **d** Linear regression of the pENTRY expression vs pDEST secretion for the DsbA E1 and Piii E5. All data was taken from shaker flask expression under different inducer concentrations at 30 °C, 14 h post induction performed in biological triplicates

The synonymous codon libraries encoding the signal peptides of interest were generated by site directed mutagenesis, to produce variants at codons 2 through to 6 using pENTRY (Additional file 1: Table S2). The theoretical library sizes ranged from 48 to 256 variants dependent on the specific signal peptides, sufficient colonies were screened to ensure 95% coverage (> 3-times theoretical size per library), using the BL21(LV2) expression strain. Hits were selected on the basis of expanded riboswitch-dependent expression control relative to the starting (WT) sequence. Strains with the selected codon-optimised and WT signal peptide sequences were treated with increasing inducer concentration to assess expression and titratability (Additional file 1: Fig. S3). All selected codon variant strains exhibited higher maximum expression compared to their respective WT. Most variants showed a modest increase of maximum expression (up to twofold), whereas the Piii-E5 variant showed the highest expression increase, 577-fold higher than the strain with the WT signal peptide (Table 1). In the absence of any inducer, all strains showed minimal fluorescence signal. Expression in the presence of only the transcriptional inducer (IPTG = 150 µM) was reduced relative to wild type for the SRP-dependent pathway, whereas the reverse was observed for SecB-dependent signal peptides. In terms of regulatory performance the strain with the Piii-E5 signal peptide exhibited the largest dynamic range both for riboswitch-dependent control (IP/I) (16-fold), and total expression control (IP/UI) (127-fold) (Fig. 2a). The strains with DsbA-E1 and yBGL2-H1 also presented good riboswitch-dependent control of expression (IP/I) of 11- fold and 13-fold, and total expression control (IP/UI) 33-fold and 60-fold respectively. This is in comparison to other inducible T7 RNAP expression systems that have been reported to display twofold expression control of secretion [41]. All strains with codon optimised signal peptide constructs were PPDA-titratable and showed improved expression and titratability compared to WT constructs indicating a good integration of the riboswitch (Additional file 1: Fig. S3).

**Table 1:** Expression performance of the BL21(LV2)-pENTRY strains containing the codon optimised signal peptide sequences

	Signal peptide	Max (IP)	IPTG (I)	Uninduced (UI)	IP/I	IP/UI	PPDA concentration (μM) at max (IP)	OD_600_ at max (IP)
SRP-dependent	DsbA WT	1629	754	64	2	26	8	1 ± 0
	DsbA E1	2152	202	65	11	33	400	18 ± 5
	yBG L2 WT	4017	1283	75	3	54	8	11 ± 10
	yBGL2 H1	4493	336	75	13	60	200	20 ± 4
SecB-dependent	Piii WT	17	21	34	1	0	400	14 ± 6
	Piii E5	9818	607	77	16	127	400	24 ± 3
	PelB WT	1297	179	74	7	18	400	21 ± 2
	PelB A5	2913	355	76	8	38	100	16 ± 6

Gene expression monitored as relative fluorescence units (RFU) over optical cell density (OD) in the absence of inducer (UI), with IPTG induction only (I) and with both IPTG and PPDA induction (IP)

Due to resource limitation and metabolic burden upon the host, higher protein production usually negatively impacts the cell density of bacterial culture [42, 43]. However, strains containing codon-optimised signal peptides DsbA-E1 and Piii-E5, both displayed increased biomass (OD_600_) and higher expression per cell (RFU/OD) than the respective strains with WT signal peptides. Indeed induction dependent inhibition of cell growth was more prominent for the WT signal peptides (Additional file 1: Fig. S3). This observation seems to indicate that the optimised signal peptides permit more efficient expression and reduced host burden. Both the SecB-dependent (Piii-E5) and the SRP-dependent (DsbA-E1) constructs, displayed significant improvements, in terms of expression and control, over their respective constructs with wild-type signal sequences (Fig. 2a). Overall the SecB-dependent Piii-E5 construct presented the highest maximum expression, best regulatory performance and biomass accumulation, while the SRP-dependent DsbA-E1 construct exhibited the best dose response profile (Additional file 1: Fig. S3). The DsbA-E1 and Piii-E5 constructs were selected and sub-cloned (pDEST) to remove the GFP fusion (“Methods” section).

### Codon optimised signal peptides permit tuneable expression and secretion of scFvβ

Expression and secretion performance of pDEST-scFvβ containing the DsbA-E1 and Piii-E5 signal peptides was assessed using *E. coli* BL21(LV2) expression strain following induction at 30 °C for 14 h (“Methods” section). Lower maximum protein production per cell (yield), expressed as mg of recombinant protein per g of dry cell weight (mg/g DCW) was achieved for the strain with the DsbA-E1 signal peptide (9 mg/g DCW) compared to the Piii-E5 (29 mg/g DCW) (Fig. 2b) (Additional file 1: Table S3). Both strains displayed excellent basal control with no detectable production of scFvβ in the absence of induction. Further, both strains displayed good riboswitch-dependent (IP/I) control of expression of 7 and 11-fold for DsbA-E1 and Piii-E5 signal peptides respectively. In terms of secretion the strain with DsbA-E1 displayed a good yield and secretion efficiency (7.6 mg/g and 81% respectively), whereas the strain with Piii-E5 displayed a slightly lower yield and poorer efficiency (5.6 mg/g and 19%), due to greater total production and retention of scFvβ in the spheroplast fraction. Addition of the inducers did not greatly compromise the biomass, with only a small reduction (15%) in final OD_600_ (Additional file 1: Table S3). Both strains displayed good riboswitch-dependent (IP/I) control of secretion of 6 and 13-fold for DsbA-E1 and Piii-E5 respectively, demonstrating that the control afforded by the system permits attenuation of scFvβ through the SecYEG translocon both via the SRP and SecB-dependent pathways (Fig. 2b).

Analysis of the half maximal effective concentration (EC_50_) indicates that the expression with DsbA-E1 (10 ± 2 μM) is saturated at higher inducer concentration compared to Piii-E5 (23 ± 6 μM). In terms of secretion both signal peptides/pathways displayed similar sensitivity/saturation (EC_50_), DsbA-E1 (7 ± 2 μM) and Piii-E5 (9 ± 6 μM) (Additional file 1: Table S3). Interestingly, this closer matching of the EC_50_ values between expression and secretion for the DsbA-E1 seems to reflect the greater degree of coordination between translation and secretion of the co-translational SRP pathway [14]. Under these conditions both signal peptides/pathways displayed similar yield, with the co-translation (SRP) pathway performing with greater secretion efficiency (Additional file 1: Table S3). To assess the utility of using the pENTRY (GOI-GFP fusion) plasmid to select signal peptide sequences with optimised codon usage for use in the final secretion pDEST plasmids, we sought to correlate induction-dependent regulatory control from strains with these plasmids (pENTRY vs. pDEST) (Fig. 2c, d). For both signal peptides expression from the scFv-GFP fusion (pENTRY) displayed linear regression coefficient (slope ~ 1) with total expression of the scFv protein (pDEST). Expression from the pENTRY also displayed close to linear coefficient with secretion pDEST for the DsbA-E1 signal peptide (slope ~ 0.8), whereas the coefficient with secretion for the Piii-E5 signal peptide was reduced (slope ~ 0.2).

### Performance of codon optimised signal peptides in the absence of translational riboswitch control

To evaluate and benchmark protein production and secretion in the *RiboTite* system compared to standard expression systems, the scFvβ gene bearing the same signal peptide sequences (DsbA E1 and Piii E5) were sub cloned into a compatible expression plasmid (pET), and expression assessed in the most commonly used T7 RNAP expression strain, BL21(DE3) (“Methods” section). Bacterial cell cultures were grown under the same conditions, and induced for 14 h at 30 °C. The non-riboswitch containing strains (BL21(DE3)-pET) produced scFvβ in yields of 22 and 12 mg/g DCW, and periplasmic secretion yields of 3.5 and 3.6 mg/g DCW for DsbA-E1 and Piii-E5 respectively, affording periplasmic secretion efficiencies of 16 and 30% (Additional file 1: Fig. S4). The final OD_600_ achieved for the BL21(DE3)-pET strains was 1.5 and 3.7 with the Piii-E5 and DsbA-E1 signal peptides, compared to OD_600_ 10 and 11 for the respective signal peptides in the BL21(LV2)-pDEST strains. This compromise in final biomass led to lower total expression and periplasmic secretion titres for scFvβ in the non-riboswitch DsbA-E1 (25.3 ± 3.6 and 4.5 ± 0.7 mg/L) and Piii-E5 strains (6.2 ± 0.5 and 1.9 ± 0.3 mg/L). This is compared to expression and secretion titres in the BL21(LV2) DsbA-E1 (36.7 ± 10.4 and 26.2 ± 7.3 mg/L) and Piii-E5 strains (101.7 ± 31.1 and 17.0 ± 0.2 mg/L) (Table 2). Regulatory control of 17 and 3-fold was observed for the DsbA-E1 and Piii-E5 signal peptide respectively in the BL21(DE3) strain. No basal expression was detected for either signal peptide in the BL21(LV2) within the western blot detection limit. This analysis was performed using a highly sensitive near infra-red fluorescent detection technique which is capable of detecting down to 50 pg of scFvβ, equivalent to 0.01 mg/L based on biomass of OD_600_ = 10. In summary the BL21(LV2) strain permitted better secretion per cell (yield), better secretion efficiency, along with better biomass accumulation than the BL21(DE3) strain. The cumulative benefits of these improvements lead to a significant improvement up to ninefold increase in scFvβ secretion titres.

**Table 2:** scFvβ expression and periplasmic secretion titres with DsbA E1 and Piii E5 signal peptides in the BL21(LV2)-pDEST and BL21(DE3)-pET28 strains

	scFvβ			
Signal peptide	DsbA-E1		Piii-E5	
BL21 strain	(LV2)	(DE3)	(LV2)	DE3
Expression titre mg/L	36.7 ± 10.4	25.3 ± 3.6	101.7 ± 31.1	6.2 ± 0.5
Induction control (max/basal)	≥ 3666	17	≥ 10,165	3
Secretion titre mg/L	26.2 ± 7.3	4.5 ± 0.7	17.0 ± 0.2	1.9 ± 0.3
OD_600_ at max	11.0 ± 0.3	3.7 ± 0.3	10.0 ± 0.5	1.5 ± 0.0

Performed in shaker flask induced for 14 h at 30 °C. No basal expression was observed in the BL21(LV2) strains, within the western blot detection limit. Induction control (max/basal) was calculated relative to the lower detection limit

### Codon optimised signal peptides permit tuneable expression and secretion of alternative scFv’s

**Fig. 3:**
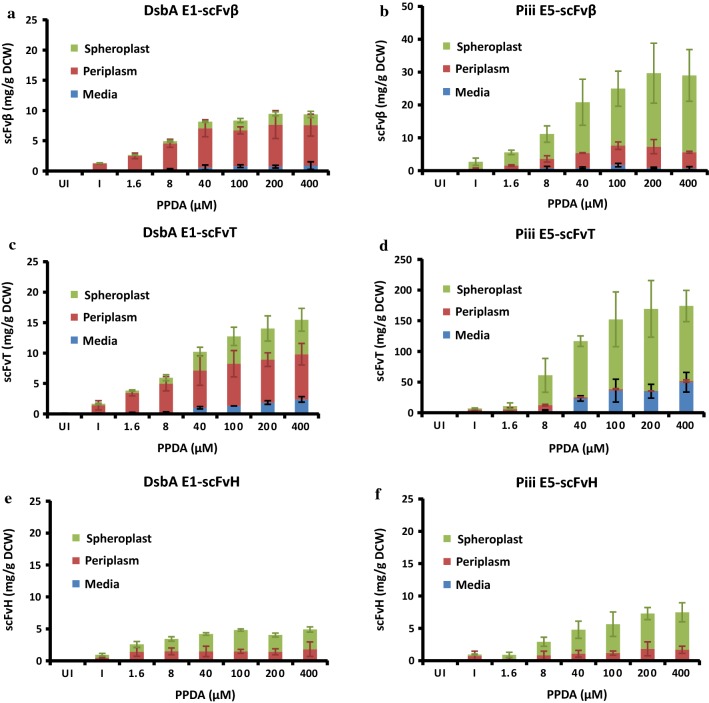
Performance of different signal peptide-scFv pDEST from shake flask expression at 30 °C, 14 h post induction. Using the DsbA E1 signal peptide sequence (**a**, **c**, **e**) and the Piii E5 signal peptide sequence (**b**, **d**, **f**) expressing scFvβ (**a**–**b**), scFvT (**c**–**d**), and scFvH (**e**–**f**). The scFv yield plotted as mg per g dry cell weight (mg/g DCW) against inducer concentrations for different scFvs, quantified from western blot analysis, from the media (M), periplasm (PP) and spheroplast (SP) fractions. Performed in the absence of inducer (UI), or with the same IPTG (I) concentration (150 μM) and increasing PPDA concentrations (1.6–400 μM). All data was taken from shaker flask expression under different inducer concentrations at 30 °C, 14 h post induction performed in biological triplicates

To explore the modularity of both the approach and the selected signal peptides, expression and secretion of alternative single chain antibody fragments, anti-histone (scFvH) [31] and anti-tetanus (scFvT) [32] was explored (Fig. 3) (Additional file 1: Table S3 and Fig. S5). In terms of total expression, the scFv’s were differently produced ranging from 5 to 16 mg/g for strains with the DsbA-E1 signal peptide to between 8 and 138 mg/g with Piii-E5. Despite this variability, rank order of scFv expression was maintained (scFvT > scFvβ > scFvH). All strains displayed riboswitch-dependent (IP/I) control of expression between 5 to 11-fold, with the Piii-E5 generally outperforming the DsbA-E1. In terms of secretion, Piii-E5-scFvT displayed the best yield but the poorest efficiency (12.8 mg/g and 9% respectively), due to the retention of scFv in the spheroplast fraction. At maximal induction secretion efficiency was highly variable but greater efficiency was observed for SRP-dependent pathway (DsbA-E1, 37–81%) compared to the SecB-dependent pathway (Piii-E5, 9–23%). The strains with DsbA-E1-scFvT and Piii-E5-scFvβ displayed the best riboswitch-dependent (IP/I) control of protein secretion of 7 and 13-fold respectively. Intriguingly, clear attenuation of scFvβ and scFvT in periplasmic fraction is observed with DsbA-E1 up to maximum of ~ 7 mg/g (Fig. 3a–c). However, above a certain level (> 4 mg/g) greater retention of scFv is observed in the spheroplast fraction, indicating a system capacity overload at these higher production levels. Similarly at higher production levels (> 6 mg/g) release of scFv into the media fraction was observed.

**Fig. 4:**
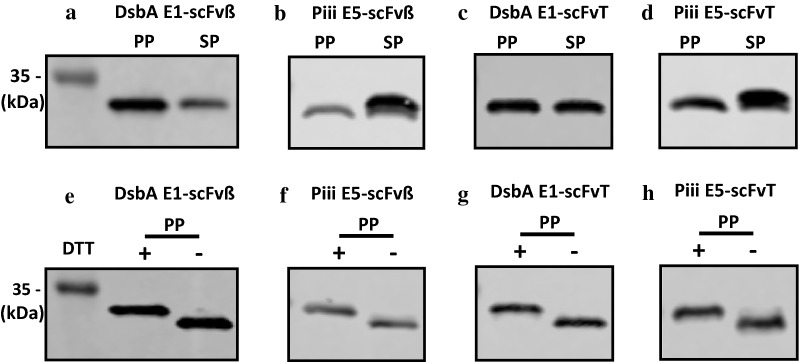
Western blot analysis of the periplasm (PP) and spheroplast (SP) samples from shaker flask induction. **a**–**d** Precursor protein processing and spheroplast retention of scFvβ and scFvT were assessed with either DsbA E1 or Piii E5 signal peptide. **e**–**h** The same periplasm fractions were also assessed for the disulfide bond formation under reducing and non-reducing conditions, with (+) or without (−) DTT

To verify proper post-secretion processing of the scFv from the higher producing constructs, scFvβ and scFvT (Fig. 2a–d), intact mass spectrometry was used (Additional file 1: Fig. S6), which showed that all scFv’s isolated from the periplasm, were correctly processed mature proteins (signal peptide absent), following correct signal peptidase-I processing. The scFvT isolated from the media fraction was also analysed by intact mass spectrometry and also validated the correct processing of the recombinant protein (Additional file 1: Fig. S7). The scFvβ and scFvT proteins were also assessed by size-exclusion chromatography coupled with multi-angle light scattering, this indicated that both were monomeric with apparent molecular mass values that correspond to the expected protein molecular weight (Additional file 1: Fig. S8). In order to further assess the precursor protein processing and spheroplast retention of scFvβ and scFvT, proteins located in the spheroplast and periplasm fractions were analysed by western blot, following SDS-PAGE using an extended running time to separate the protein forms (Fig. 4a–d). Analysis of DsbA E1-scFvβ indicates that the target protein located in both the spheroplast and periplasm fractions has the same retention time (Fig. 4a), and the same is also observed for DsbA E1-scFvT (Fig. 4c). In contrast, analysis of Piii E5-scFvβ and Piii E5-scFvT (Fig. 4b, d) indicates the spheroplast fractions contain two species, the processed scFv and presumably the precursor, with the precursor being the dominant species. Due to the small difference in molecular weight between the DsbA and Piii signal peptides (1.99 vs. 2.16 kDa), the processed and precursor forms for DsbA-dependent constructs should, in principle, be resolved by SDS-PAGE, as per the Piii-dependent constructs. On this basis, it appears possible that the spheroplast fraction for the DsbA E1-dependent samples contains the mature processed protein. The same periplasm fractions for scFvβ and scFvT were also assessed for the disulfide bond formation under reducing and non-reducing conditions (Fig. 4e–h). The faster migration of non-reduced samples is due to their more compact structure, which indicates correct disulfide bond formation of the scFv’s within the periplasm. Finally the scFvβ and scFvH isolated from the periplasm fraction were also analysed for binding activity to β-galactosidase and histone substrates respectively (“Methods” section), and displayed binding affinity values (Additional file 1: Fig. S9 and Fig. S10) comparable to literature values [44].

### Expression and secretion control performance is maintained under fed-batch fermentation

**Fig. 5:**
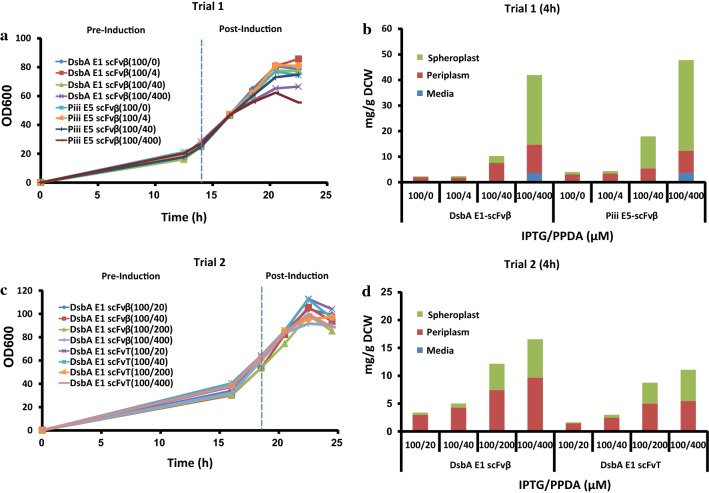
Fed-batch fermentation cell growth, scFv protein yield and location. **a**, **b** Trial 1—scFvβ expressed with DsbA-E1 or Piii-E5 signal peptides samples collected at 4 h post-induction. **c**, **d** Trial 2—scFvβ or scFvT expressed with DsbA-E1 signal peptide samples collected at 4 h post-induction. The scFv yields plotted against inducer concretion (IPTG/PPDA) fixed IPTG (100 µM) and increasing PPDA concentrations (0, 4, 20, 40, 200, 400 µM) with scFv quantified from western blot analysis in the media (M), periplasm (PP) and spheroplast (SP) fractions. Protein production quantification was performed in technical triplicates

Fed-batch fermentation experiments were performed on the ambr250 multi-parallel bio-reactor system. Initial trials focused on scFvβ production with both the DsbA-E1 and Piii-E5 signal peptides in the BL21(LV2)-pDEST strain (Fig. 5a, b). Following inoculation bioreactor cultures were grown in batch mode until a sharp dissolved oxygen increase, used as an indicator of nutrient limitation, then an exponential glucose feed was initiated to achieve a specific growth rate (μ = 0.2) until the end of the fermentation (22.5 h). The cultures were induced at OD_600_ = 20–30, with fixed IPTG (100 µM) and different PPDA (0, 4, 40, 400 µM) concentrations. Following addition of inducers all cultures grew with similar growth kinetics for the first 2 h, whilst between 4 and 6 h post-induction the culture with the highest concentration of inducers displayed reduced biomass accumulation (Fig. 5a). At 8 h post-induction the final biomass varied from OD_600_ = 80 to 50 dependent on the inducer concentration. This inverse trend between inducer concentration and final biomass was consistent with cell viability (Additional file 1: Fig. S11). Samples for 4 h post induction (18 h) were analysed for protein production and secretion (Fig. 5b, Table 3) (Additional file 1: Table S4). The highest expression level was achieved with the highest concentration of inducers (IPTG: 100 µM, PPDA: 400 µM). Similar yields and titres were observed for both strains with the Piii-E5 (51 mg/g DCW, 996 mg/L) and DsbA-E1 (43 mg/g DCW, 859 mg/L) signal peptides. No expression ‘leak’ was observed prior to induction (14 h). Induction with IPTG-only led to basal protein production in both the strains (2–3 mg/g DCW, 36–66 mg/L). Addition of PPDA (400 µM) resulted in riboswitch-dependent expression control of 27-fold and 17-fold for the DsbA-E1 and Piii-E5 respectively (Fig. 5b). In terms of secretion similar yields and titres were observed, with the DsbA-E1 (12 mg/g DCW, 248 mg/L) slightly outperformed by the Piii-E5 (14 mg/g DCW, 269 mg/L). Secretion efficiency was slightly higher for the DsbA-E1 (29%) than the Piii-E5 (27%), at the highest inducer concentration. At low inducer concentration secretion efficiency increases significantly up to ~ 80% for both DsbA E1 and Piii E5 (Fig. 5b). The scFvβ isolated from the periplasm fraction were also analysed for binding activity to β-galactosidase substrates (Methods), and displayed binding affinity values (Additional file 1: Fig. S9 and Fig. S10) comparable to literature values [44].

**Table 3:** Expression, secretion titers and secretion efficiency under fed-batch fermentation

		Expressed	Secreted	
		mg/L	mg/L	%
Trial 1 (4 h)	DsbA E1-ScFvβ	859 ± 199	248 ± 65	29
	Piii E5-ScFvβ	996 ± 117	269 ± 29	27
Trial 2 (4 h)	DsbA E1-ScFvβ	572 ± 72	352 ± 73	62
	DsbA E1-ScFvT	392 ± 10	210 ± 34	54
Trial 2 (6 h)	DsbA E1-ScFvβ	436 ± 41	230 ± 28	53
	DsbA E1-ScFvT	417 ± 33	219 ± 38	53

To further explore the secretion productivity seen with the DsbA-E1 signal peptide, another fermentation experiment was performed using the DsbA-E1 signal peptide with scFvβ and scFvT (Fig. 5c, d). As very tight control of expression in the absence induction was observed in the initial trial, but reduced biomass accumulation was also observed for induction times ≥ 4 h, a modified growth/induction strategy was implemented. The batch-fed transition was maintained as before (“Methods” section), but cultures were induced later at OD_600_ = 55–65, with fixed IPTG (100 µM) and different PPDA (20, 40, 200, 400 µM) concentrations. Prior to induction no leaky expression was detected (Additional file 1: Table S5). Following addition of inducers all cultures grew with similar growth kinetics for the first 4 h, attaining biomass of OD_600_ = 95–110 at 4 h post-induction, further induction time led to a plateau in growth and drop in biomass, possibly due to dilution with the continuous feed (Fig. 5c). Good viability was observed for all induction conditions and times (Additional file 1: Fig. S11).

**Fig. 6:**
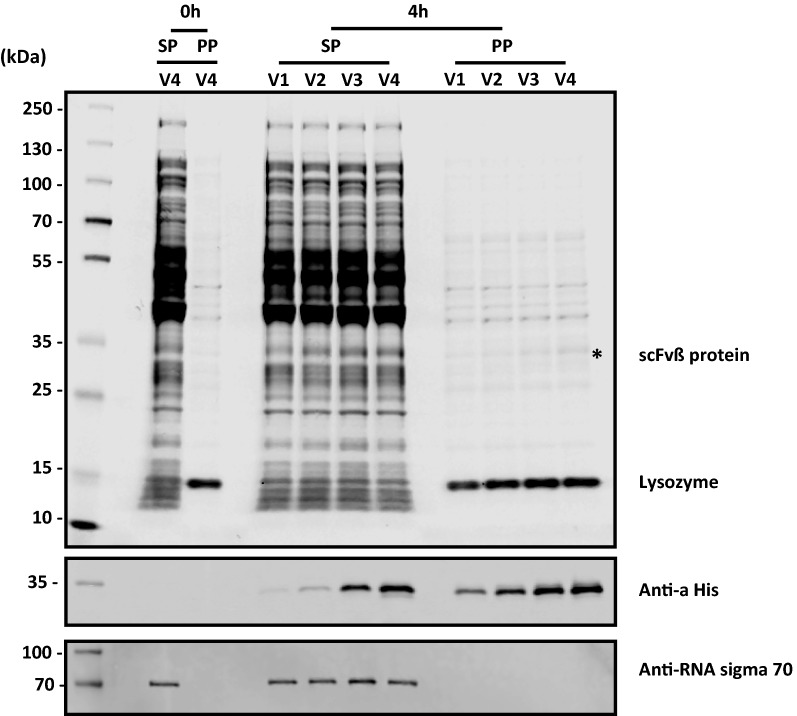
SDS-PAGE and western blot analysis of Trial 2 fermentation samples. Coomassie stained SDS PAGE (upper pannel) and western blot analysis were performed on both spheroplast (SP) and periplasm (PP) samples of the pDEST-DsbA E1-scFvβ from 0 h and 4 h post-induction. The induction conditions were IPTG (100 µM) and increasing concentrations of PPDA (4, 40, 200 and 400 µM). The scFvβ protein was detected using the Anti-α His antibody. The *E. coli* anti-RNAP σ70 was used as a control and the signal was only detected in the SP cellular fractions demonstrating the correct cell fractionation procedure. Lysozyme (labelled) was used for the fractionation procedure

The scFvβ and scFvT from the periplasm fraction were purified and analysed using intact mass spectrometry to confirm that the protein is correctly processed by cleavage of the signal peptide (Additional file 1: Fig. S7). To demonstrate the correct cell fractionation procedure an indicative coomassie stained SDS-PAGE and western blot analysis is shown (Fig. 6). Western blot analysis against the cytoplasmic specific marker (sigma 70) indicates correct fractionation, due to the absence of signal in the periplasmic fractions. Precursor protein processing and spheroplast retention of scFvβ and scFvT were assessed by western blot, following SDS-PAGE (Additional file 1: Fig. S12). Consistent with the shake flask analysis, both DsbA E1-scFvβ and DsbA E1-scFvT from spheroplast fractions were composed of only one species. The same periplasm fractions were also assessed for disulfide bond formation under reducing and non-reducing conditions; which demonstrated disulfide bond formation for both scFvβ and scFvT isolated from the periplasm (Additional file 1: Fig. S12). The highest scFv production was achieved with the highest concentration of inducers (IPTG: 100 µM, PPDA: 400 µM), with higher yields and titres observed for the scFvβ (17 mg/g DCW, 572 mg/L), compared to scFvT (11 mg/g DCW, 392 mg/L) at 4 h post-induction (Fig. 5d) (Additional file 1: Table S5 and Fig. S13). Production levels at 6 h for scFvβ decreased slightly (14 mg/g DCW, 436 mg/L), whereas scFvT increased slightly (13 mg/g DCW, 417 mg/L) (Additional file 1: Fig. S13 and Table S6). In terms of secretion yields and titres, scFvβ had maximal production at 4 h post induction (11 mg/g DCW, 352 mg/L), whereas scFvT had a maximal production at 6 h post induction (7 mg/g DCW, 219 mg/L) (Table 3) (Additional file 1: Table S5 and Table S6). Both displayed similar secretion efficiency (53–62%) at the highest inducer concentration. Additionally secretion efficiency was modulated under riboswitch-dependent control, achieving up to 90% efficiency at lower inducer concentrations (Fig. 5d).

Comparing between the two fermentation trials the total production yield and titre of DsbA-E1-scFvβ were higher (2.5-fold and 1.5-fold respectively) in the initial fermentation trial (Additional file 1: Table S4 and S5). However, the second trial displayed enhanced secretion efficiency (29% vs. 62%), in addition to enhanced biomass accumulation (OD_600_ 57 vs. 90) and cell viability (CFU/mL/OD 2.4 × 10^9^ vs. 8.5 × 10^10^), this led to a similar titre for scFvβ secretion from both trials (248 vs. 352 mg/L). However, secreted scFv was all contained within the periplasm in trial 2, whereas trial 1 exhibited substantial leakage (25%) across the outer membrane to the media fraction.

## Discussion

Here we have shown that use of the multi-layered gene expression control system, *RiboTite,* in combination with codon optimised signal peptide sequences, permits attenuation of recombinant expression and periplasmic secretion of single chain antibody fragments (scFvs). In this study we employed the use of an orthogonal translation riboswitch control element (ORS), which releases and sequesters a RBS in the presence and absence of the small molecule inducer (PPDA) [23]. A modified T7 RNAP-dependent *E. coli* expression strain BL21(LV2), was developed and benchmarked for expression and control against BL21(DE3). This system uses two small molecule inducers (IPTG and PPDA) that operate at the transcriptional and translation level respectively, controlling expression of both the T7 RNAP and the gene of interest [23]. This new strain displayed excellent riboswitch-dependent control (> 40-fold), and extremely large small molecule-dependent (IPTG + PPDA) control of expression (> 1200-fold), which as far as we are aware is an unprecedented induction dynamic range for T7 RNAP-dependent expression systems (Additional file 1: Table S1).

The exemplar gene of interest, coding for the single chain antibody fragment anti-β-galactosidase (scFvβ) [30], was initially expressed as a GFP fusion protein (pENTRY) to permit the rapid selection of signal peptide sequences from a synonymous codon library (Fig. 1). Codon usage is an important feature for optimal heterologous gene expression [45], and a large number of algorithms have been developed to optimize codon usage for recombinant genes [46–49]. The 5′ coding region of genes for secreted proteins are known to be enriched with ‘non-optimal’ or rare codons [50–52]. Clustering of non-optimal codons in the N-terminal region of the signal peptide is believed to slow the rate of translation and allow efficient engagement with secretion apparatus [51]. An alternative to this ‘ramp’ hypothesis is derived from the observation that non-optimal codons have a higher proportion of A-T pairs, affording transcripts with reduced local secondary structure [37, 53, 54]. For secretion of recombinant proteins, non-optimal codon usage in the signal peptide sequence has been shown to positively impact protein folding and export [33, 55]. Optimal integration of the orthogonal riboswitch (ORS) into the 5′UTR recently demonstrated that codon selection is determined by structural features rather than codon rarity [40]. Here in this study the codon selection method permitted functional context-dependent integration of orthogonal riboswitch in the 5′UTR to afford a broad, inducer-dependent, dynamic range of gene expression control. The small sample size (n = 8) in this current study did not permit thorough statistical analysis of codon and mRNA folding metrics for the identified signal peptide sequences to support or exclude either the ramp or structural hypothesis. The selected clones included codon optimised signal peptide sequences for both the SecB-dependent (Piii-E5) and SRP-dependent (DsbA-E1) pathways, permitting good riboswitch dependent (up to 16-fold), and total (up to 127-fold) control over eGFP reporter gene expression.

Removal of the reporter fusion afforded pDEST, which demonstrated absolute control of basal expression in absence of induction and excellent dynamic range control of gene expression and secretion (Fig. 2b, Table 2). Under batch shake flask conditions scFvβ was less well-expressed and secreted with the DsbA-E1 signal peptide, however this difference in performance was reduced under fed-batch fermentation conditions (Fig. 5, Table 3). Correlation of inducible expression performance between fusion and non-fusion constructs (pENTRY vs. pDEST) indicated that dynamic range of expression control for both showed excellent linearity and good regression coefficients (slope) validating the approach and utility the pENTRY screen to select for optimal codon variants (Fig. 2c, d). Interestingly, the iterative (post-translocation) mechanism of SecB-dependent pathway was clearly demonstrated by a small regression coefficient (shallow slope) between expression of the GFP-fusion and secretion performance of the non-fusion. In the same manner comparison of the secretion efficiency and the dose–response curves (EC_50_), indicated the better coordination and coupling between expression and secretion for the SRP-dependent pathway (Additional file 1: Table S3).

When compared against the classical T7 RNAP-dependent inducible promoter/operator *E. coli* expression strain, BL21(DE3), the *RiboTite* system permitted greater control over scFvβ expression and secretion and displayed enhanced secretion titre (up to ninefold) (Additional file 1: Fig. S4). Exchange of the scFvβ for alternative antibody fragments anti-histone (scFvH) [31], and anti-tetanus (scFvT) [32] was performed, and these related proteins display amino acid and nucleotide sequence identity down to 87%. Regulatory control was maintained for all scFvs, however, expression yields were very sensitive to the gene of interest (Fig. 3). This observation was consistent with previous reports which indicate that variability within the complementarity-determining region (CDR) of antibody fragments significantly affects production yields [56]. For each scFv protein expressed, the DsbA-E1 gave lower total expression compared to the Piii-E5, but better secretion efficiency for both shake flask and fed-batch fermentation experiments. For all samples analysed, scFv isolated from the periplasm were correctly processed with disulfide bond formation and activity. For all batch and fed-batch experiments, retention of target scFv was observed in the spheroplast fraction. Product retained in the spheroplast for Piii-E5 (SecB) was predominately precursor protein, which had not been translocated or cleaved, indicative of an overload of the secretion pathway. However, product retained in the spheroplast for DsbA-E1 (SRP) appears to be processed mature scFv, based on identical SDS-PAGE retention to scFv from the periplasm, indicating scFv protein insolubility in the periplasm, and/or overload of the periplasmic folding capacity. Previous studies have also shown that use of SRP-dependent signal peptide sequences increased secretion yield and efficiency of recombinant proteins, by avoiding premature cytoplasmic folding associated with the SecB-dependent pathway [10, 57].

The ability to secrete recombinant proteins into the *E. coli* periplasmic compartment is limited by the periplasm size and the secretion capacity of the cell. The smaller periplasmic compartment accounts for less than 20% of the total cell volume [58]. Depending on the strain, signal peptide and protein of interest used for secretion, there is a certain threshold of the protein amount that can be exported into the periplasmic compartment. For recombinant human proinsulin, an upper secretion limit of 7.2 mg/g DCW was previously reported [5]. Previous studies on the secretion of scFv’s under both batch and fed-batch conditions have reported between 50 and 90 mg/L [10, 59], higher values have been reported, but periplasmic titres above > 400 mg/L resulted in significant cell lysis [60]. Here, in this study under fed-batch fermentation a periplasmic secretion yield for scFvβ of 14 mg/g DCW with the Piii-E5, and 12 mg/g DCW with DsbA-E1 was observed (Additional file 1: Table S4). Exceeding a specific limit from each condition led to accumulation of protein in the media fraction. In our studies intact mass spectrometry analysis showed that the scFv protein detected in the media culture is processed correctly, the signal peptide was cleaved from the recombinant protein, indicating the protein was translocated across the inner membrane via SecYEG, and was released across the outer membrane (Additional file 1: Fig. S6). It has been recognised that recombinant protein secretion can lead to release of the protein into the cultivation media [25, 61]. The exact mechanism is not yet known, but outer membrane protein and lipid composition have been shown to be altered during prolonged fermentation conditions [62, 63]. It is well also known that the SecYEG-dependent secretion apparatus can easily become overloaded [17, 41, 64]. To overcome this careful optimisation is required to match recombinant expression rate to the secretion capacity of the host to maximise translocation efficiency. In previous studies we demonstrated, with a closely related strain, that the *RiboTite* system produced recombinant GFP fourfold slower (RFU/OD/hr) than the classically *E. coli* T7 RNAP-dependent strain, BL21(DE3), and the rate of expression could be reduced a further eightfold at lower inducer concentrations [23]. In this study the slower expression kinetics of the *RiboTite* system and the ability to attenuate the expression rate, permitted a range of expression rates to be assessed and matched to host secretion rate, to maximise secretion efficiency (Fig. 3).

In this study, the *RiboTite* system produced industrially relevant titres of scFv under fed-batch fermentation conditions. Further improvements in secretion titres could be achieved by co-expression with periplasmic chaperones and helpers. Indeed co-expression of molecular chaperones has been reported to be favourable to increase secretion of recombinant proteins by correct protein folding and/or promoting disulfide-bond formation [3, 8, 9]. Specifically for scFv expression, co-expression of Skp chaperone [59, 65, 66], FkpA peptidyl-prolyl isomerase [59, 67], and the disulfide bond isomerase DsbC [68, 69] have been shown to improve recombinant protein solubility and increase titres.

## Conclusion

We demonstrate that tuning gene expression, and therefore protein secretion with the *RiboTite* system is a viable approach for secretion of recombinant proteins. Codon optimisation of the signal peptide sequences allowed integration of the orthogonal riboswitch to permit fine-tuning of protein production. The *RiboTite* system permits (i) robust control over basal expression in absence of induction, and (ii) finely-tuned control over expression; to avoid overload of the Sec-dependent secretion pathway. Under fed-batch fermentation protein production and secretion titres of up to 1.0 g/L, and 0.35 g/L respectively were achieved, whilst cell viability and biomass accumulation were maintained. High product titre, quality, and activity were achieved irrespective of the Sec-dependent pathway employed, although greater secretion efficiency was observed with the SRP pathway.

Increasing host secretion efficiency and productivity is an important cost consideration for the manufacture of recombinant antibody fragments. Enhanced protein production capability can facilitate the transition of candidate therapeutic proteins towards the clinic by limiting manufacturing failure during early stage development. Additionally reduced manufacturing costs could lessen the financial burden upon healthcare providers, and permit more equitable global access to protein-based therapeutic medicines.

## Methods

### Strains, plasmids, inducers and equipment

Expression strains: *E. coli* BL21 (DE3) (NEB). BL21(IL3) strain with parental BL21 genetic background, but contains an integrated orthogonal riboswitch that controls T7RNAP; BL21(LV2) similar to the BL21 (IL3), but lacks kanamycin resistance and has the *lacI*^*q*^ gene instead of *lacI* in an opposite orientation with an additional operator (O3) between the *lacI*^*q*^ and the promoter; and K12(LV2) similar with BL21(LV2) but with parental *E. coli* K-12 W3310 genetic background. The IgG single chain antibody fragment genes (anti-β-galactosidase scFv13R4, anti-histone, and anti-tetanus toxin single-chain Fv) were synthesised by GeneART (Thermo Fisher) and sub-cloned into pENTRY and pDEST vectors, containing the kanamycin resistance marker. For strain benchmarking, the pETORS-eGFP expression vector [23] was used. For the expression in BL21(DE3) stain, the pET28 vector (no-riboswitch control), with the signal peptide and the scFv13R4 were used. For secretion constructs, synthetic DNA containing the riboswitch and signal peptide sequences (DsbA WT, PelB WT, Piii WT and yBGL2 WT) synthesized by GeneART (Thermo Fisher), and cloned upstream of the scFv gene by restriction digest and ligation via NdeI/SpeI sites. Inducers: IPTG (Isopropyl β-D-1-thiogalactopyranoside) (Sigma), PPDA (Pyrimido[4,5-d]pyrimidine‐2,4‐diamine) (Peakdale Molecular). A CLARIOstar Microplate Reader (BMG) was used to measure the eGFP fluorescence and cell density (OD_600_) for intact cells.

### Construction of BL21(LV2) and K12(LV2) strains of *E. coli*

To build on the *RiboTite* system for periplasmic secretion purposes, the BL21(IL3) strain [23] was modified to generate the BL21(LV2) strain for tighter secretion of recombinant proteins into the bacterial periplasm. A cassette was constructed in using the pZB insertion plasmid (containing chloramphenicol expression cassette flanked by two dif sites [70], modified by the addition of regions of homology to the araD and araC genes). Relative to the insertion cassette of the BL21(IL3) strain here the LacI was switched to *lacI*^*q*^ and the orientation was inverted. The modified cassette was amplified by PCR, and inserted by homologous recombination into the genome of *E. coli* BL21 (*F*^–^ *ompT gal dcm lon hsdSB (rB*^–^*mB*^–^*) [malB*^+^*]K*-*12(λS))*, within the *araC*-*D* locus, with pSIM18 [71] to generate BL21(LV2). The same cassette was also inserted into the *E. coli* strain K12 W3110 (F^−^ lambda^−^ INV(rrnD-rrnE) rph-1), to generate K12(LV2).

### Bacterial cell culture

All cell cultures were grown in TB media (2.7% yeast extract, 4.5% glycerol, 1.3% Bactotryptone) supplemented with 0.2% glucose. The cell culture for codon and strain selection assays were also grown in LB media (0.5% yeast extract, 0.5% NaCl, 1% Bactotryptone) with addition of 0.2% glucose. Plasmids were selected using ampicillin (100 µg/mL) or kanamycin (50 µg/mL), all purchased from Sigma. Cultures were inoculated directly from freshly plated recombinant colonies. For strain selection, pre-cultures were grown at 37 °C with shaking (180 rpm) to an OD_600_ = 0.3–0.4, and then transferred in 96-well deep-well plates for induction. Cultures were induced for either 3 h at 37° C or 14 h at 30 °C with shaking at 1000 rpm (Stuart microtitre plate shaker incubator). To measure fluorescence, 96-well black clear bottom plates (Greiner) were used, with measurements at an Excitation λ = 470 nm/Emission λ = 515 nm. For shake flask expression, pre-cultures were grown at 37 °C with shaking (180 rpm) to reach logarithmic growth phase (OD_600_ ~ 0.8) and then transferred for induction in 125 mL shaker flasks. The volume of culture induced was 25 mL and cells were left un-induced (UI), induced with 150 µM IPTG (I) and a combination of inducers (150 µM IPTG with varying PPDA concentrations). After induction cultures were grown at 30 °C for up to 20 h for pENTRY and at 14 h for pDEST with shaking at 210 rpm.

### Selection of signal peptides with synonymous codons

Mutagenesis was performed as per the manufacturers (NEB) protocol using Phusion HF DNA Polymerase. Mutagenic libraries of the pENTRY template were generated by PCR mutagenesis with primers randomized at the wobble position at codons 2 to 6. Dependent on the codon degeneracy of each specific amino acid, the appropriately randomised nucleotide base (Y, R, N) was incorporated in the positions within the mutagenic primer, corresponding to the 3rd nucleotide for each codon, permitting generation of a synonymous codon library. The product was DpnI treated to remove the template (37 °C, 4 h), and transformed into Top10 F’ competent *E. coli* cells. Individual colonies (N > 10) were picked and screened to confirm complete template removal and library diversity. Colonies were screened to ensure 95% coverage (threefold) of the theoretical library size, variants were selected on the basis of expanded riboswitch (PPDA) dependent control.

### Fractionation of *E. coli*

Cultures were grown as described in “Bacterial cell cultures”—shake flask section. After specific times post induction, ODV 10 (OD_600_ * mL = 10) were collected by centrifugation (6000 g, 15 min, 4 °C) and the pellet was resuspended in 250 μL Buffer 1 (100 mM Tris–acetate pH 8.2, 500 mM Sucrose, 5 mM EDTA), followed by addition of lysozyme (0.16 mg/mL) and Milli-Q water (250 μL). Cells were left on ice for 5 min and then MgSO_4_ (20 mM) was added to stabilise the spheroplasts. The periplasm (supernatant) fractions were collected by centrifugation, while the spheroplasts (pellet) were washed once with Buffer 2 (50 mM Tris–acetate pH 8.2, 250 mM Sucrose, 10 mM MgSO_4_) and resuspended in Buffer 3 (50 mM Tris–acetate pH 8.2, 2.5 mM EDTA, 0.1% Sodium Deoxycholate and 250 U/μL Benzonase). Spheroplasts were lysed by freezing at − 20 °C overnight and thawing at room temperature prior to being analysed. The media and cell fractions were stored at 4 °C short-term or at − 20 °C for long-term storage. All fractions were prepared from biological triplicates.

### Western blot analysis

The media, periplasm and spheroplast fractions were analysed by western blot, using an infrared imager LI-COR Odyssey Sa. Samples were re-suspended in SDS-PAGE loading buffer (Thermo Fisher), supplemented with 50 mM dithiothreitol, and boiled for 10 min. Samples were diluted to be within the linear range protein standard curve (below). Equal volumes of sample were loaded, separated by SDS-PAGE to confirm and quantify protein amounts by western blot. The membrane was then blocked with phosphate buffered saline (PBS) containing 5% skimmed milk for 20 min (~ 55 rpm, room temperature). The His-tagged scFv protein was detected with mouse monoclonal Anti-His antibody (Pierce, 1:3000 in PBS 5% milk) and IRDye 680RD donkey anti-mouse IgG (LI-COR, 1:20,000 in PBST 5% milk). The protein bands were visualised at 700 nm with the Odyssey Imaging System (LI-COR) and the signal intensity were quantified with the Image Studio 5.0 Software for densitometry analysis. Protein quantification was performed using purified recombinant scFv protein standard curves (between 50 pg to 120 ng). All data were measured in biological triplicates.

### scFv binding assay

The activity assay was performed using a Bio-Dot Device (Bio-Rad). A pre-wet 0.2 µm nitrocellulose membrane (Amersham Hybond) was placed in the apparatus and the membrane was rehydrated with PBS. The substrates, β-galactosidase or and core histone mix (Sigma) and the negative control (bovine serum albumin) were added onto the membrane diluted in Buffer 1 (0.5 ng/dot). The membrane was left to dry by gravity flow. A serial dilution of the periplasmic fraction containing scFv13R4 or scFvT in Buffer 1, were then added to the membrane. The membrane was left again to dry by gravity flow. A vacuum was applied to the apparatus to remove any excess liquid. The membrane was taken from the apparatus and was blocked for 20 min with 5% milk PBS (50 rpm, room temperature). His-tagged scFv13R4 or scFvT protein was detected as described above (“Western blot analysis” section). The signal intensity was quantified with the Image Studio 5.0 Software for densitometry analysis and GraphPad Prism 7 was used to for curve fitting using a four-parameter logistic function. All data were measured in biological triplicates.

### Fed batch fermentation

Starter cultures were grown overnight in 25 mL of LB with 0.2% glucose and 50 µg·mL^−1^ kanamycin at 30 °C. Overnight cultures were used to inoculate 50 mL of LB with 0.2% glucose and 50 µg·mL^−1^ kanamycin in a 250 mL baffled shake flask which was incubated at 30 °C at 200 rpm until an OD_600_ of between 2 and 4.

Fed-batch fermentations used the Ambr^®^ 250 modular (Sartorius Stedim) which comprises 250 mL single-use bioreactors. Fermentations started with 150 mL of batch medium and 100 mL of feed. The batch medium was from [72] and comprised batch salts (14 g·L^−1^ (NH_4_)_2_SO_4_, 5.5 g·L^−1^ glucose monohydrate, 20 g·L^−1^ Bacto™ yeast extract, 2 g·L^−1^ KH_2_PO_4_, 16.5 g·L^−1^ K_2_HPO_4_, 7.5 g·L^−1^ citric acid, 1.5 mL·L^−1^ concentrated H_3_PO_4_ and 0.66 mL·L^−1^ PPG 2000) and additions (34 mL·L^−1^ trace elements solution (comprising 3.36 g·L^−1^ FeSO_4_·7H_2_O, 0.84 g·L^−1^ ZnSO_4_·7H_2_O, 0.18 g·L^−1^ MnCl_2_·4H_2_O, 0.25 g·L^−1^ Na_2_MoO_4_·2H_2_O, 0.12 g·L^−1^ CuSO^4^·5H_2_O, 0.36 g·L^−1^ H_3_BO_3_ and 48 mL·L^−1^ concentrated H_3_PO^4^), 10 mL·L^−1^ 1 M MgSO_4_·7H_2_O, 2 mL·L^−1^ 1 M CaCl_2_·2H_2_O and 1 mL·L^−1^ 50 mg·mL^−1^ kanamycin stock). The feed solution comprised 220 g·L^−1^ glucose monohydrate (Trial 1—FBF) or 440 g·L^−1^ glucose monohydrate (Trial 2—FBF), 30 mL·L^−1^ 1 M MgSO_4_·7H_2_O, and 1 mL·L^−1^ 50 mg·mL^−1^ kanamycin. Batch salts were sterilised by autoclaving. All other culture medium components were filter sterilised and added to the fermentation vessels before use. The pH was maintained at 6.8 using 10% NH_4_OH and 1 M HCl. Polypropylene glycol (PPG 2 000) was used as antifoam. The dissolved oxygen (DOT) was maintained at above 20% when possible, using cascade control (increasing the stirrer speed followed by an increase in the air flow rate, and if not sufficient, by addition of O_2_). Bioreactors were inoculated to an OD_600_ of 0.1. Exponential feeding was used according to Eq. 1.

1 $$ F = \left( {\frac{1}{S}} \right) \times \left( {\frac{\mu }{{Y_{XS} }} + m} \right) \times X_{0 } \times e^{\mu t} $$

where *F* is the feed rate in L·h^−1^, *S* is the substrate concentration in the feed (depending on the fermentation run, 220 g·L^−1^ or 440 g·L^−1^ glucose monohydrate), *μ* is the required specific growth rate (0.2 h^−1^), Y_*XS*_ is the yield coefficient (0.365 g biomass per g glucose), *m* is the maintenance coefficient (0.0468), X_*0*_ is the biomass in g at the start of the feed and *t* is time. The feed was started when the DO increased, indicating nutrient limitation.

### Cell viability assay (CFU)

Culture samples taken post-induction were serially diluted in PBS and plated onto LB agar to evaluate cell culturability, used as an indication of cell viability. LB agar plates were incubated at 37 °C overnight.

### Data processing and statistical analysis

Data was processed and analysed using Microsoft Excel, GraphPad Prism7 and OriginPro 8.5.1. Each data point used for analysis was from three biological experimental repeats and was used for fitting a logistic growth curve. The EC_50_ value represents the amount of PPDA needed to achieve half of the maximum induction response. Error bars represent calculated standard deviations. For western blot quantification, the Image Studio 5.0 Software was used for densitometry analysis. A calibration curve was constructed using 3 up to 6 scFv standards. Data was fitted using linear regression into a straight line and the linear equation from the scFv calibration curve was used to normalise and convert the western blot sample into ng of protein. The measured OD_600_ were used to normalise and calculate the mg/g of dry cell weight. Dry cell weight was determined, by collecting culture in dry pre-weighed 2 mL tubes; the samples were centrifuged 10 min at 6000 g, cell pellets were dried at 100 °C for 48 h and tubes re-weighed, replicate values were used to determine an OD to g DCW conversion factor (0.35 mg/mL). Subsequently, the dry cell weight of the *E. coli* cell was calculated as the OD_600_ multiplied by conversion factor (0.35 mg/mL). Linear regression was employed to analyse correlation at 30 °C induction between pENTRY and pDEST (secretion and expression). The relationship between western blot data and dot blot data was again investigated by linear regression. Semilog regression analysis evaluated the relationship between pENTRY and pDEST. Pearson’s correlation coefficient and the best-fit line were calculated. P < 0.05 was considered statistically significant.

### Protein purification

All scFv proteins expressed have a hexa-histidine tag to allow purification by standard immobilized metal affinity chromatography (IMAC) using HisPur™ Ni–NTA Resin (ThermoFisher). The proteins used as standard for western blot quantification were purified using whole cell lysates from cell cultures expressing the genes of interest (“Bacterial cell culture”). Cell pellets were collected by centrifugation (9000*g* for 30 min) and resuspended in lysis buffer (50 mM Tris HCl pH 7.5, 500 mM NaCl, 10 mM imidazole) supplemented with EDTA-free protease inhibitor (Roche), DNAase (10U/mL) and lysozyme (1 mg/mL). Cells were sonicated and the supernatant was collected by high-speed centrifugation (42,000*g*). Supernatant was incubated with the Ni–NTA beads for at least 1 h at 4 °C. Protein was washed 3 × times with lysis buffer and then eluted with 100 mM imidazole. Protein was then concentrated using 5000 MWCO Vivaspin centrifugal units and then dialysed with dialysis buffer (25 mM Tris HCl pH 7.5, 50 mM NaCl). Protein purity was assessed by SDS-PAGE and its concentration determined using NanoDrop 2000 Spectophotometer and extinction coefficient. Protein was stored at − 80 °C.

### Intact mass spectrometry

A 1200 series Agilent LC was used to inject 5 µL of sample into 5% acetonitrile (0.1% formic acid) and desalted inline. This was eluted over 1 min by 95% acetonitrile. The resulting multiply charged spectrum was analysed by an Agilent QTOF 6510, and deconvoluted using Agilent Masshunter Software.

### Size-exclusion chromatography coupled with multi-angle light scattering (SEC-MALS) analysis

Samples were loaded onto a Superdex 75 26/600 column (GE healthcare) pre-equilibrated in protein dialysis buffer (25 mM Tris pH 7.5, 50 mM NaCl) running at a flow rate of 0.75 mL/min. Samples were analysed using a DAWN Wyatt HeliosII 18-angle laser photometer, with an additional Wyatt QELS detector. This was coupled to a Wyatt Optilab rEX refractive index detector and the molecular mass moments, polydispersity, and concentrations of the resulting peaks were analysed using Astra 6.1 software (Wyatt, Santa Barbara, USA).

## Additional file

**Additional file 1.** Additional figures and tables.
