# Supplementary material for: Tuning recombinant protein expression to match secretion capacity
Source: Microb Cell Fact. 2018 Dec 22;17:199. doi: 10.1186/s12934-018-1047-z (PMC6303999; doi:10.1186/s12934-018-1047-z)
Supplement: Supplementary file 1 — Additional file 1. Additional figures and tables. [file 12934_2018_1047_MOESM1_ESM.pptx]

## Slide 1
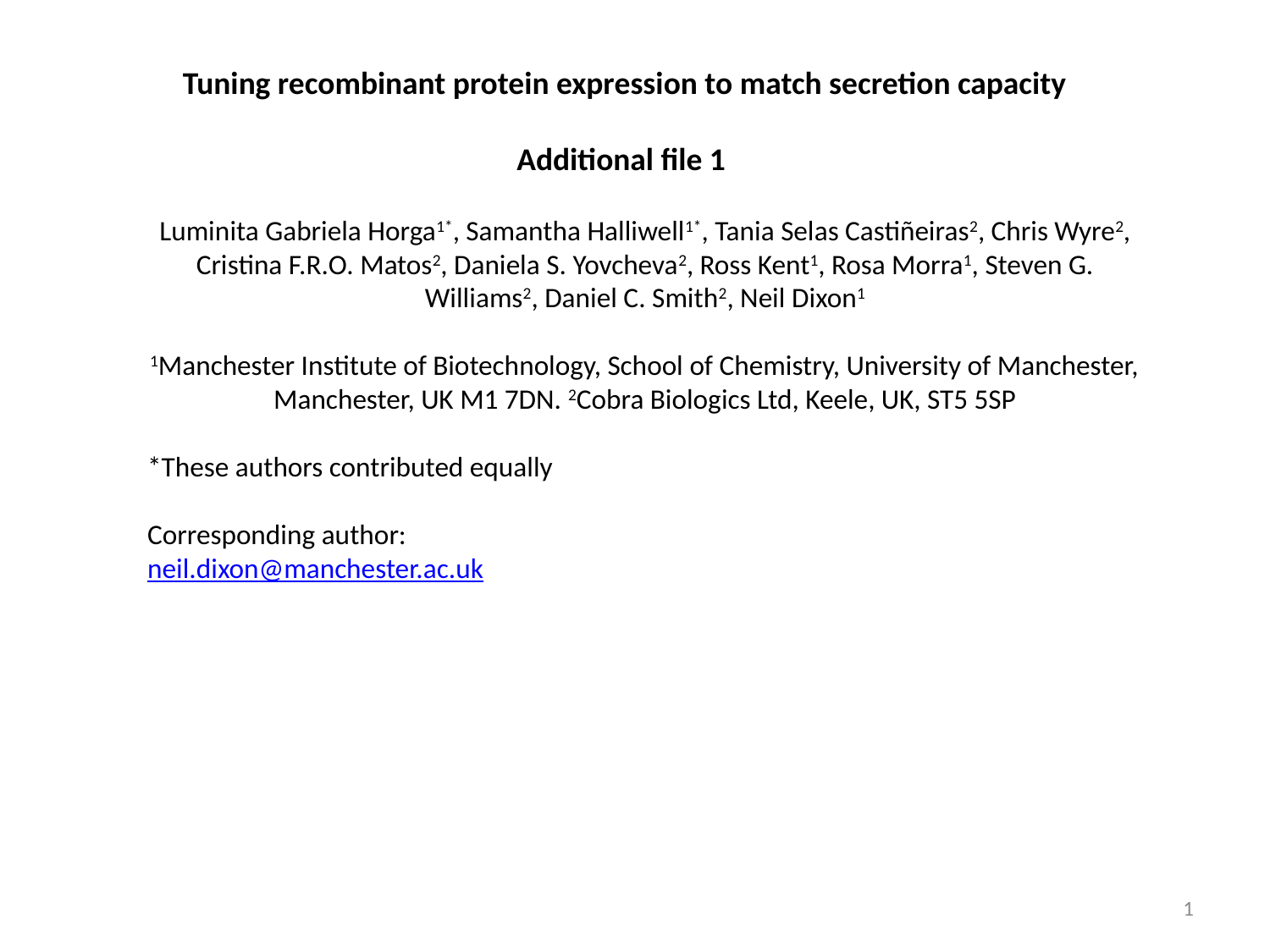

Tuning recombinant protein expression to match secretion capacity
Additional file 1
Luminita Gabriela Horga1*, Samantha Halliwell1*, Tania Selas Castiñeiras2, Chris Wyre2, Cristina F.R.O. Matos2, Daniela S. Yovcheva2, Ross Kent1, Rosa Morra1, Steven G. Williams2, Daniel C. Smith2, Neil Dixon1
1Manchester Institute of Biotechnology, School of Chemistry, University of Manchester, Manchester, UK M1 7DN. 2Cobra Biologics Ltd, Keele, UK, ST5 5SP
*These authors contributed equally
Corresponding author:
neil.dixon@manchester.ac.uk
1

## Slide 2
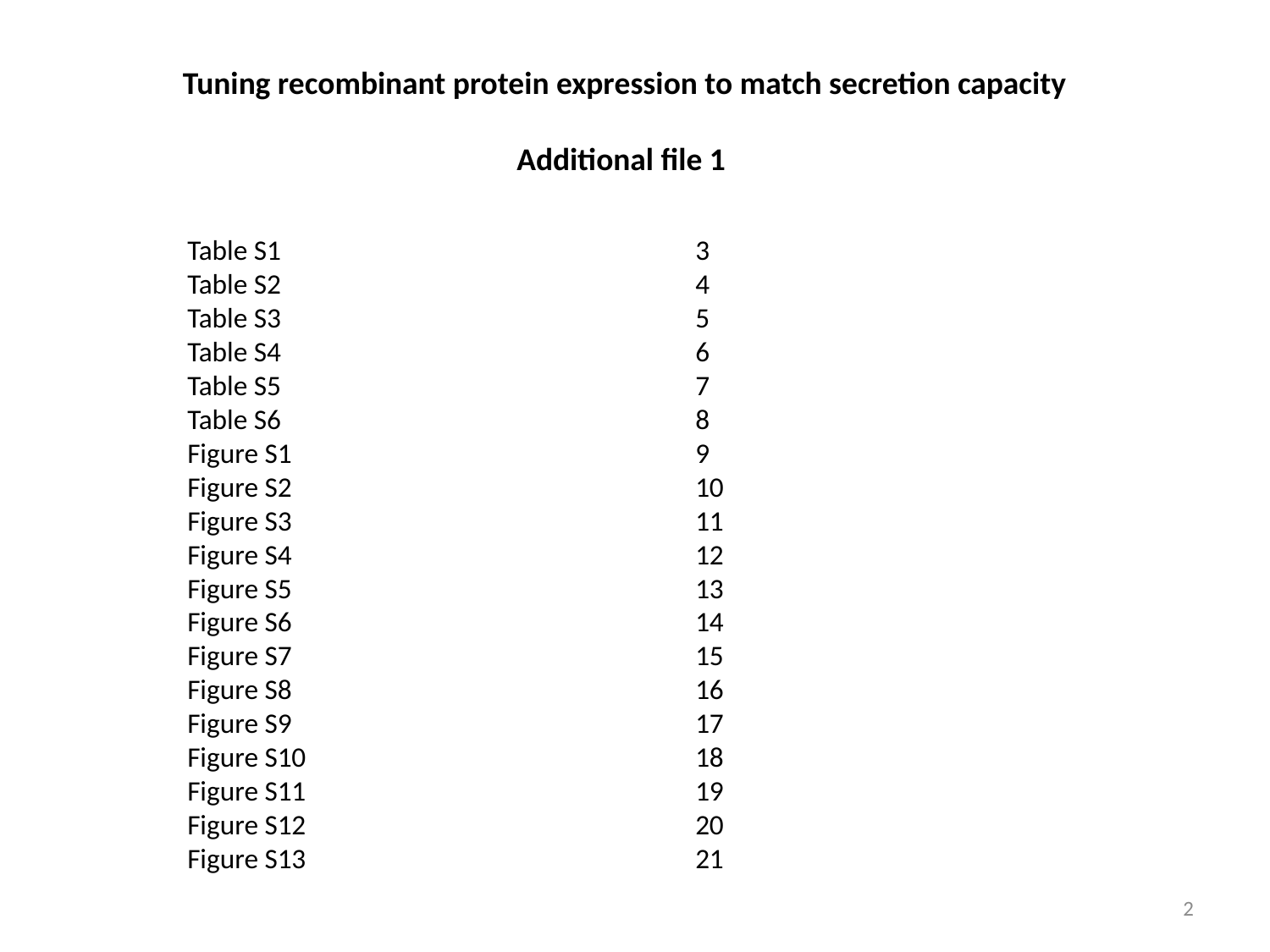

Tuning recombinant protein expression to match secretion capacity
Additional file 1
Table S1				3
Table S2				4
Table S3				5
Table S4				6
Table S5				7
Table S6				8
Figure S1				9
Figure S2				10
Figure S3				11
Figure S4				12
Figure S5				13
Figure S6				14
Figure S7				15
Figure S8				16
Figure S9				17
Figure S10				18
Figure S11				19
Figure S12				20
Figure S13				21
2

## Slide 3
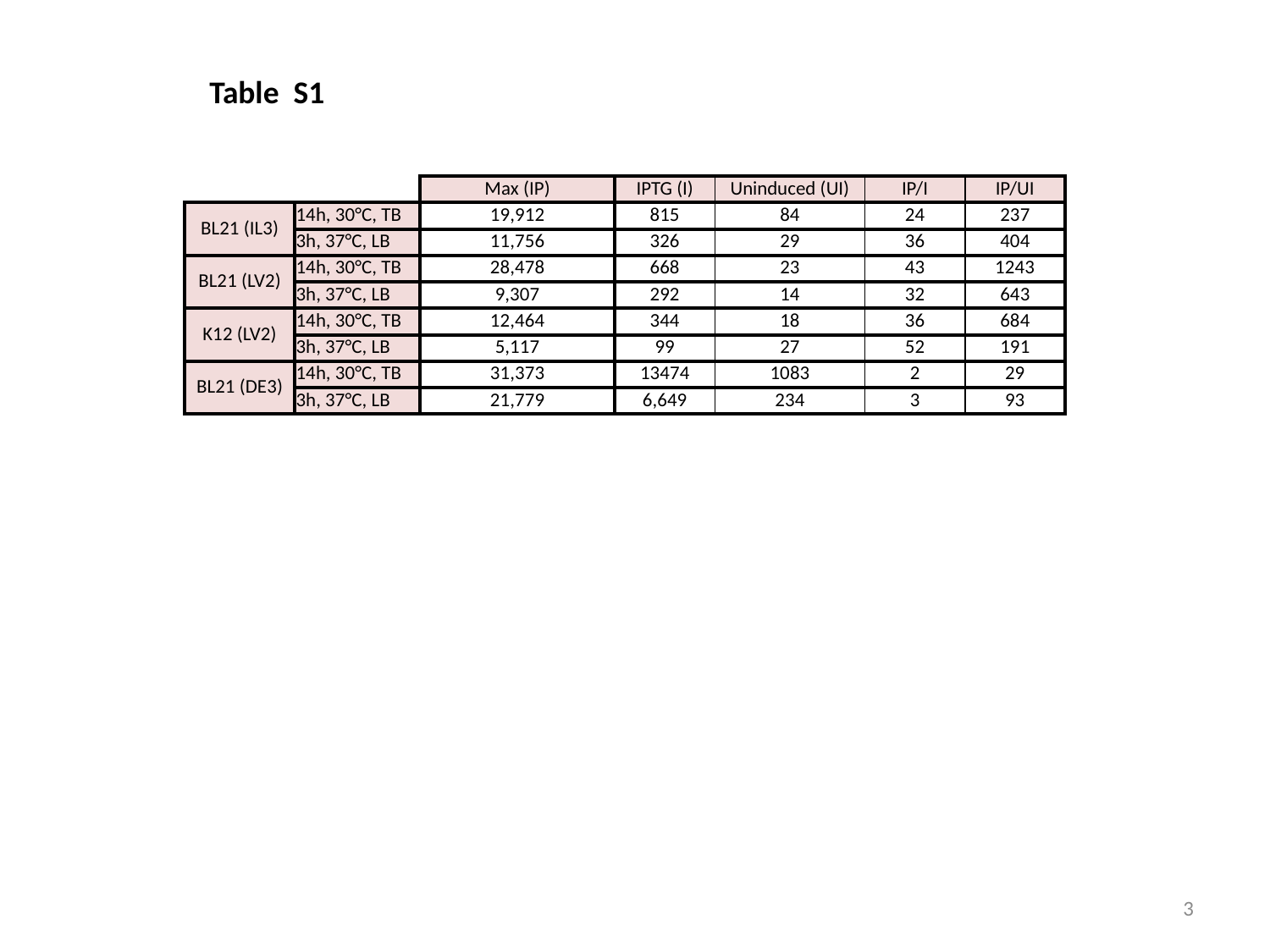

Table S1
| | | Max (IP) | IPTG (I) | Uninduced (UI) | IP/I | IP/UI |
| --- | --- | --- | --- | --- | --- | --- |
| BL21 (IL3) | 14h, 30°C, TB | 19,912 | 815 | 84 | 24 | 237 |
| | 3h, 37°C, LB | 11,756 | 326 | 29 | 36 | 404 |
| BL21 (LV2) | 14h, 30°C, TB | 28,478 | 668 | 23 | 43 | 1243 |
| | 3h, 37°C, LB | 9,307 | 292 | 14 | 32 | 643 |
| K12 (LV2) | 14h, 30°C, TB | 12,464 | 344 | 18 | 36 | 684 |
| | 3h, 37°C, LB | 5,117 | 99 | 27 | 52 | 191 |
| BL21 (DE3) | 14h, 30°C, TB | 31,373 | 13474 | 1083 | 2 | 29 |
| | 3h, 37°C, LB | 21,779 | 6,649 | 234 | 3 | 93 |
3

## Slide 4
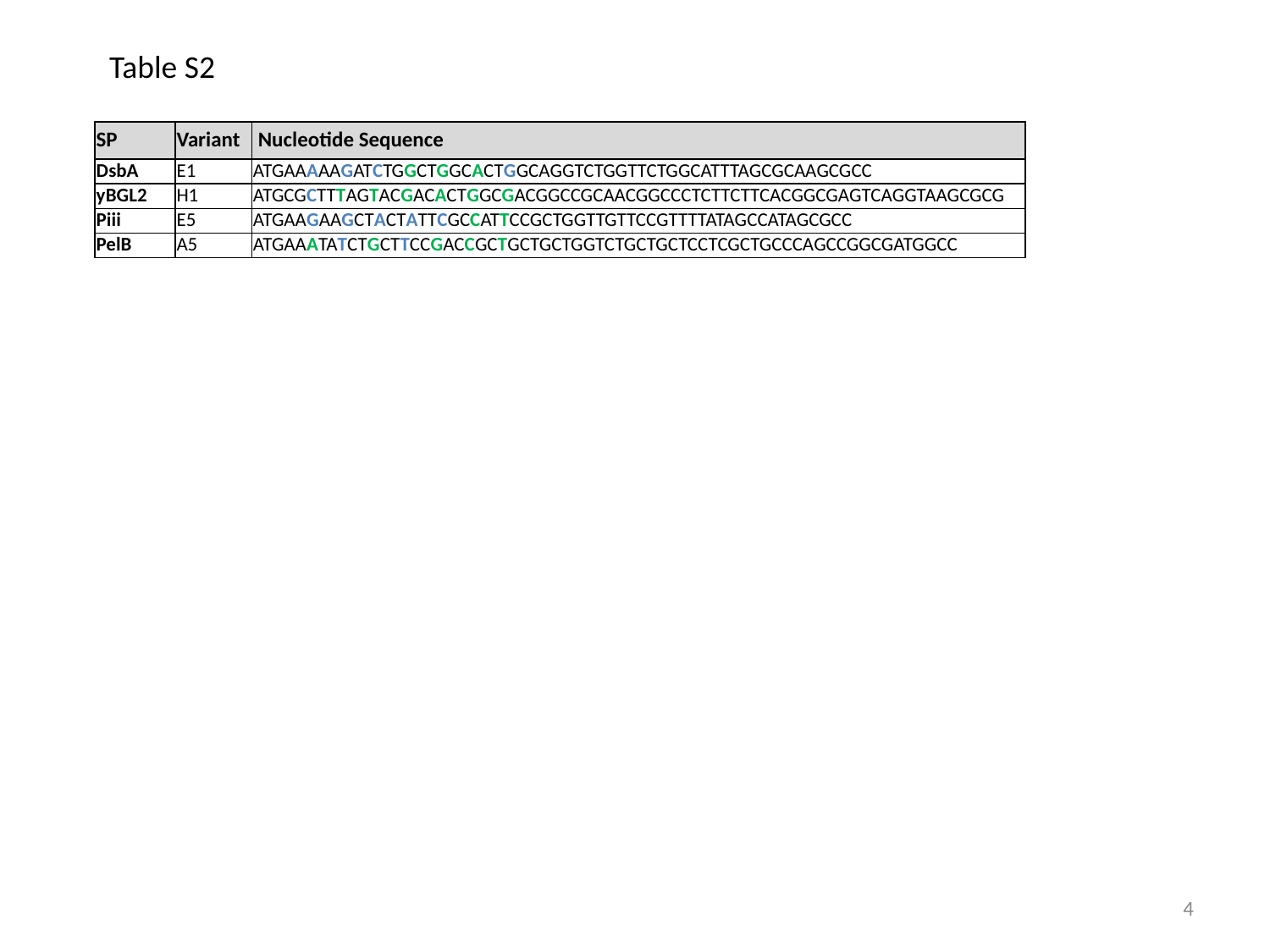

Table S2
| SP | Variant | Nucleotide Sequence |
| --- | --- | --- |
| DsbA | E1 | ATGAAAAAGATCTGGCTGGCACTGGCAGGTCTGGTTCTGGCATTTAGCGCAAGCGCC |
| yBGL2 | H1 | ATGCGCTTTAGTACGACACTGGCGACGGCCGCAACGGCCCTCTTCTTCACGGCGAGTCAGGTAAGCGCG |
| Piii | E5 | ATGAAGAAGCTACTATTCGCCATTCCGCTGGTTGTTCCGTTTTATAGCCATAGCGCC |
| PelB | A5 | ATGAAATATCTGCTTCCGACCGCTGCTGCTGGTCTGCTGCTCCTCGCTGCCCAGCCGGCGATGGCC |
4

## Slide 5
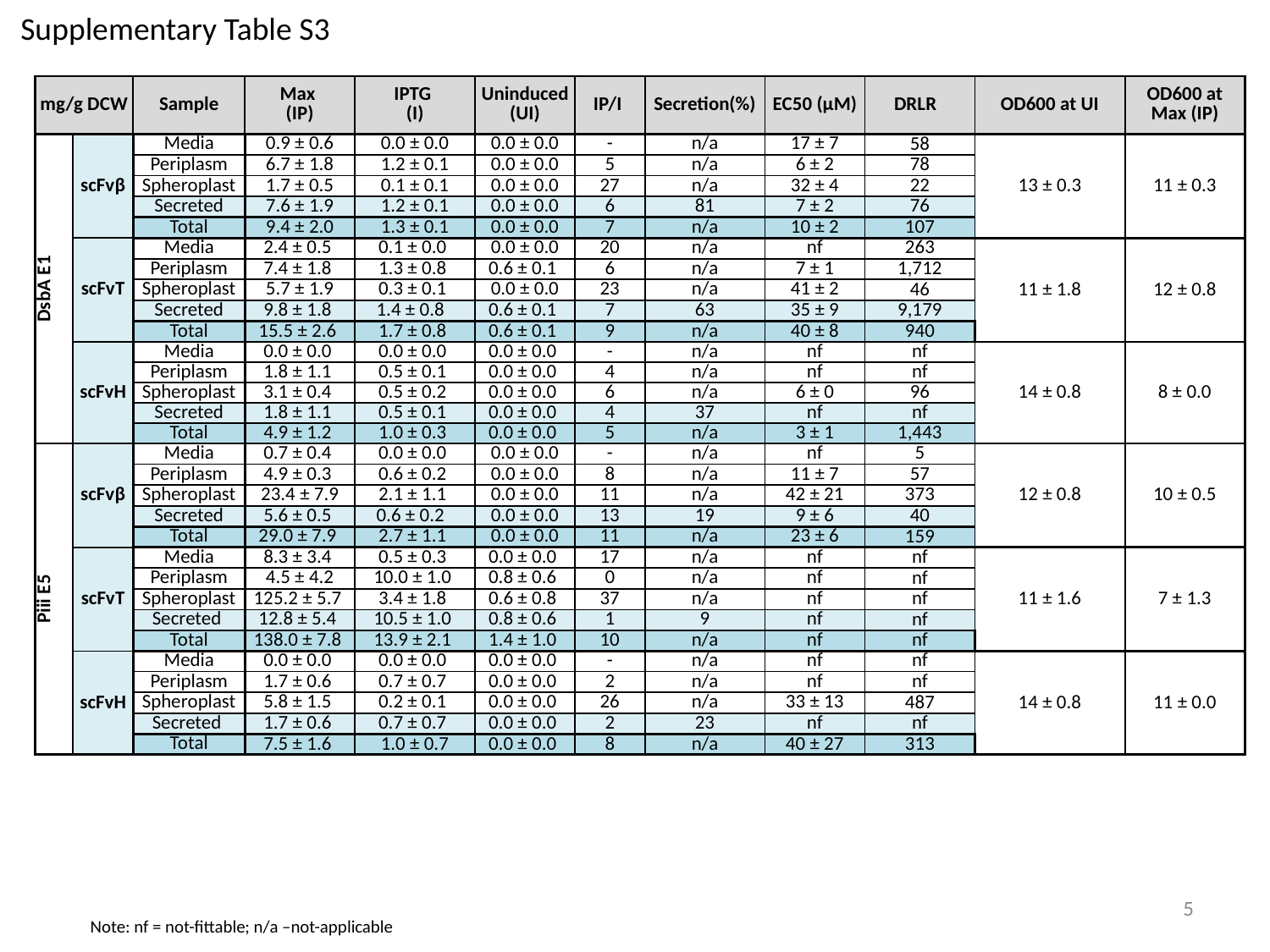

Supplementary Table S3
| mg/g DCW | | Sample | Max (IP) | IPTG (I) | Uninduced (UI) | IP/I | Secretion(%) | EC50 (µM) | DRLR | OD600 at UI | OD600 at Max (IP) |
| --- | --- | --- | --- | --- | --- | --- | --- | --- | --- | --- | --- |
| DsbA E1 | scFvβ | Media | 0.9 ± 0.6 | 0.0 ± 0.0 | 0.0 ± 0.0 | - | n/a | 17 ± 7 | 58 | 13 ± 0.3 | 11 ± 0.3 |
| | | Periplasm | 6.7 ± 1.8 | 1.2 ± 0.1 | 0.0 ± 0.0 | 5 | n/a | 6 ± 2 | 78 | | |
| | | Spheroplast | 1.7 ± 0.5 | 0.1 ± 0.1 | 0.0 ± 0.0 | 27 | n/a | 32 ± 4 | 22 | | |
| | | Secreted | 7.6 ± 1.9 | 1.2 ± 0.1 | 0.0 ± 0.0 | 6 | 81 | 7 ± 2 | 76 | | |
| | | Total | 9.4 ± 2.0 | 1.3 ± 0.1 | 0.0 ± 0.0 | 7 | n/a | 10 ± 2 | 107 | | |
| | scFvT | Media | 2.4 ± 0.5 | 0.1 ± 0.0 | 0.0 ± 0.0 | 20 | n/a | nf | 263 | 11 ± 1.8 | 12 ± 0.8 |
| | | Periplasm | 7.4 ± 1.8 | 1.3 ± 0.8 | 0.6 ± 0.1 | 6 | n/a | 7 ± 1 | 1,712 | | |
| | | Spheroplast | 5.7 ± 1.9 | 0.3 ± 0.1 | 0.0 ± 0.0 | 23 | n/a | 41 ± 2 | 46 | | |
| | | Secreted | 9.8 ± 1.8 | 1.4 ± 0.8 | 0.6 ± 0.1 | 7 | 63 | 35 ± 9 | 9,179 | | |
| | | Total | 15.5 ± 2.6 | 1.7 ± 0.8 | 0.6 ± 0.1 | 9 | n/a | 40 ± 8 | 940 | | |
| | scFvH | Media | 0.0 ± 0.0 | 0.0 ± 0.0 | 0.0 ± 0.0 | - | n/a | nf | nf | 14 ± 0.8 | 8 ± 0.0 |
| | | Periplasm | 1.8 ± 1.1 | 0.5 ± 0.1 | 0.0 ± 0.0 | 4 | n/a | nf | nf | | |
| | | Spheroplast | 3.1 ± 0.4 | 0.5 ± 0.2 | 0.0 ± 0.0 | 6 | n/a | 6 ± 0 | 96 | | |
| | | Secreted | 1.8 ± 1.1 | 0.5 ± 0.1 | 0.0 ± 0.0 | 4 | 37 | nf | nf | | |
| | | Total | 4.9 ± 1.2 | 1.0 ± 0.3 | 0.0 ± 0.0 | 5 | n/a | 3 ± 1 | 1,443 | | |
| Piii E5 | scFvβ | Media | 0.7 ± 0.4 | 0.0 ± 0.0 | 0.0 ± 0.0 | - | n/a | nf | 5 | 12 ± 0.8 | 10 ± 0.5 |
| | | Periplasm | 4.9 ± 0.3 | 0.6 ± 0.2 | 0.0 ± 0.0 | 8 | n/a | 11 ± 7 | 57 | | |
| | | Spheroplast | 23.4 ± 7.9 | 2.1 ± 1.1 | 0.0 ± 0.0 | 11 | n/a | 42 ± 21 | 373 | | |
| | | Secreted | 5.6 ± 0.5 | 0.6 ± 0.2 | 0.0 ± 0.0 | 13 | 19 | 9 ± 6 | 40 | | |
| | | Total | 29.0 ± 7.9 | 2.7 ± 1.1 | 0.0 ± 0.0 | 11 | n/a | 23 ± 6 | 159 | | |
| | scFvT | Media | 8.3 ± 3.4 | 0.5 ± 0.3 | 0.0 ± 0.0 | 17 | n/a | nf | nf | 11 ± 1.6 | 7 ± 1.3 |
| | | Periplasm | 4.5 ± 4.2 | 10.0 ± 1.0 | 0.8 ± 0.6 | 0 | n/a | nf | nf | | |
| | | Spheroplast | 125.2 ± 5.7 | 3.4 ± 1.8 | 0.6 ± 0.8 | 37 | n/a | nf | nf | | |
| | | Secreted | 12.8 ± 5.4 | 10.5 ± 1.0 | 0.8 ± 0.6 | 1 | 9 | nf | nf | | |
| | | Total | 138.0 ± 7.8 | 13.9 ± 2.1 | 1.4 ± 1.0 | 10 | n/a | nf | nf | | |
| | scFvH | Media | 0.0 ± 0.0 | 0.0 ± 0.0 | 0.0 ± 0.0 | - | n/a | nf | nf | 14 ± 0.8 | 11 ± 0.0 |
| | | Periplasm | 1.7 ± 0.6 | 0.7 ± 0.7 | 0.0 ± 0.0 | 2 | n/a | nf | nf | | |
| | | Spheroplast | 5.8 ± 1.5 | 0.2 ± 0.1 | 0.0 ± 0.0 | 26 | n/a | 33 ± 13 | 487 | | |
| | | Secreted | 1.7 ± 0.6 | 0.7 ± 0.7 | 0.0 ± 0.0 | 2 | 23 | nf | nf | | |
| | | Total | 7.5 ± 1.6 | 1.0 ± 0.7 | 0.0 ± 0.0 | 8 | n/a | 40 ± 27 | 313 | | |
5
Note: nf = not-fittable; n/a –not-applicable

## Slide 6
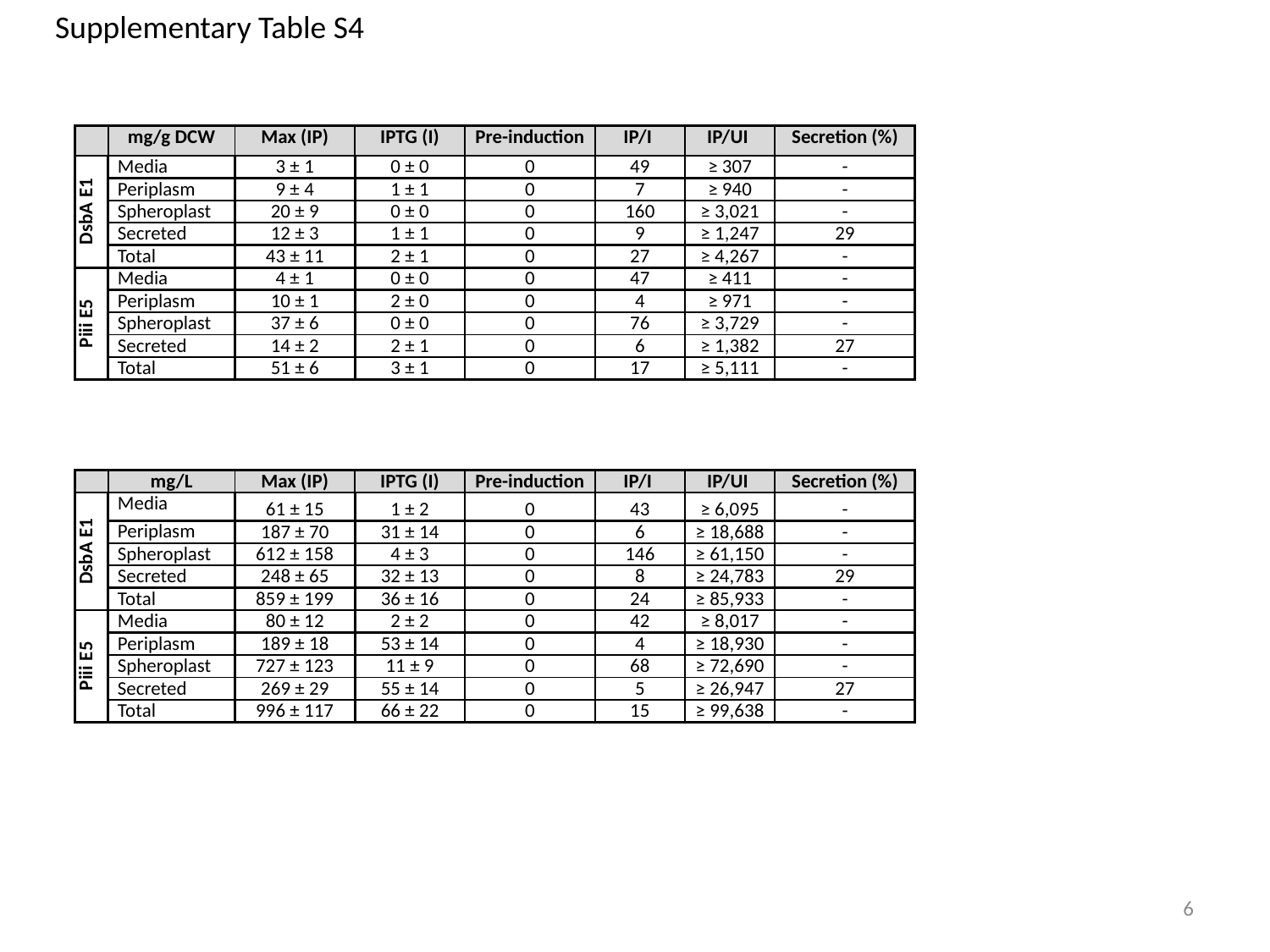

Supplementary Table S4
| | mg/g DCW | Max (IP) | IPTG (I) | Pre-induction | IP/I | IP/UI | Secretion (%) |
| --- | --- | --- | --- | --- | --- | --- | --- |
| DsbA E1 | Media | 3 ± 1 | 0 ± 0 | 0 | 49 | ≥ 307 | - |
| | Periplasm | 9 ± 4 | 1 ± 1 | 0 | 7 | ≥ 940 | - |
| | Spheroplast | 20 ± 9 | 0 ± 0 | 0 | 160 | ≥ 3,021 | - |
| | Secreted | 12 ± 3 | 1 ± 1 | 0 | 9 | ≥ 1,247 | 29 |
| | Total | 43 ± 11 | 2 ± 1 | 0 | 27 | ≥ 4,267 | - |
| Piii E5 | Media | 4 ± 1 | 0 ± 0 | 0 | 47 | ≥ 411 | - |
| | Periplasm | 10 ± 1 | 2 ± 0 | 0 | 4 | ≥ 971 | - |
| | Spheroplast | 37 ± 6 | 0 ± 0 | 0 | 76 | ≥ 3,729 | - |
| | Secreted | 14 ± 2 | 2 ± 1 | 0 | 6 | ≥ 1,382 | 27 |
| | Total | 51 ± 6 | 3 ± 1 | 0 | 17 | ≥ 5,111 | - |
| | mg/L | Max (IP) | IPTG (I) | Pre-induction | IP/I | IP/UI | Secretion (%) |
| --- | --- | --- | --- | --- | --- | --- | --- |
| DsbA E1 | Media | 61 ± 15 | 1 ± 2 | 0 | 43 | ≥ 6,095 | - |
| | Periplasm | 187 ± 70 | 31 ± 14 | 0 | 6 | ≥ 18,688 | - |
| | Spheroplast | 612 ± 158 | 4 ± 3 | 0 | 146 | ≥ 61,150 | - |
| | Secreted | 248 ± 65 | 32 ± 13 | 0 | 8 | ≥ 24,783 | 29 |
| | Total | 859 ± 199 | 36 ± 16 | 0 | 24 | ≥ 85,933 | - |
| Piii E5 | Media | 80 ± 12 | 2 ± 2 | 0 | 42 | ≥ 8,017 | - |
| | Periplasm | 189 ± 18 | 53 ± 14 | 0 | 4 | ≥ 18,930 | - |
| | Spheroplast | 727 ± 123 | 11 ± 9 | 0 | 68 | ≥ 72,690 | - |
| | Secreted | 269 ± 29 | 55 ± 14 | 0 | 5 | ≥ 26,947 | 27 |
| | Total | 996 ± 117 | 66 ± 22 | 0 | 15 | ≥ 99,638 | - |
6

## Slide 7
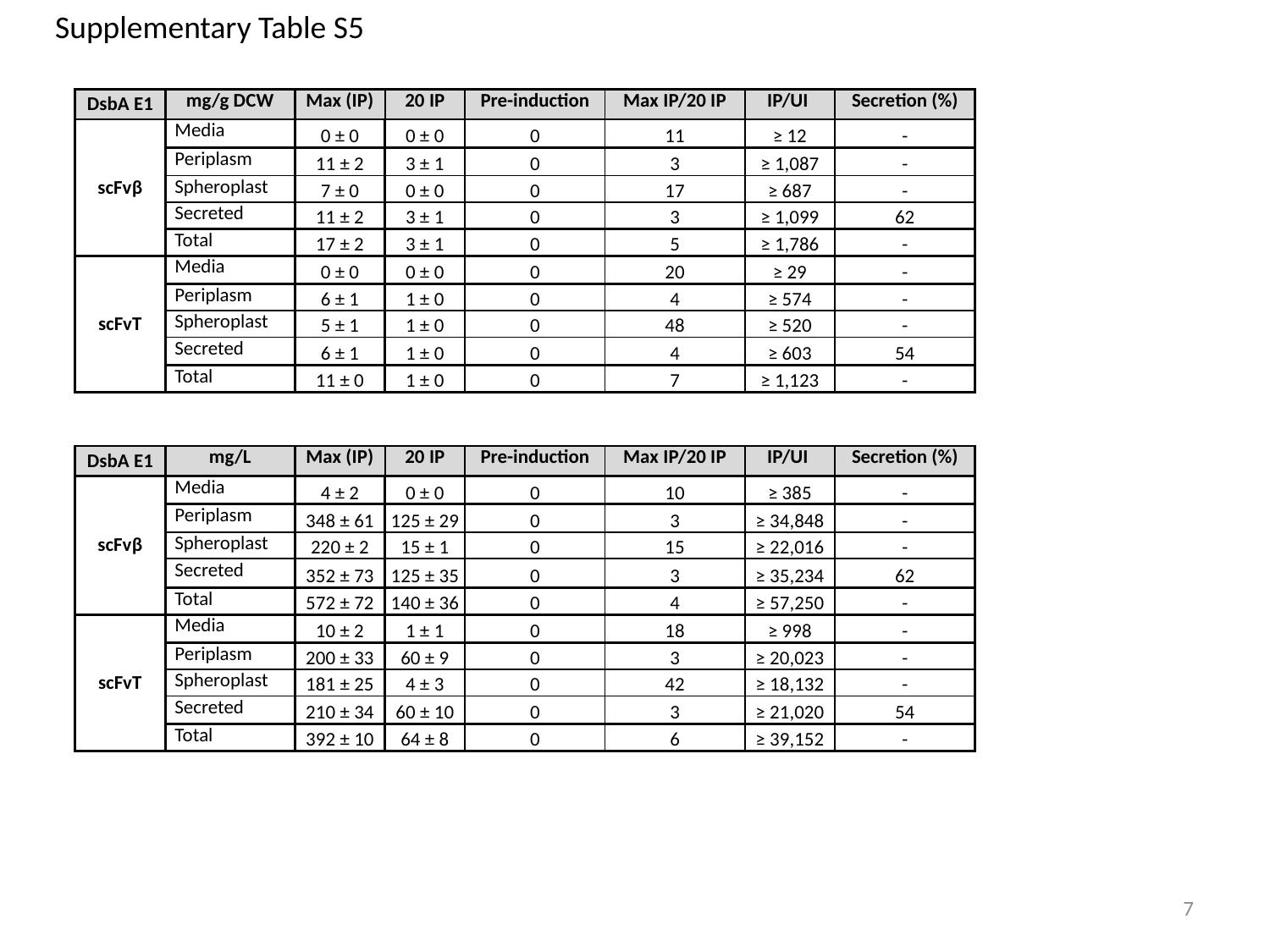

Supplementary Table S5
| DsbA E1 | mg/g DCW | Max (IP) | 20 IP | Pre-induction | Max IP/20 IP | IP/UI | Secretion (%) |
| --- | --- | --- | --- | --- | --- | --- | --- |
| scFvβ | Media | 0 ± 0 | 0 ± 0 | 0 | 11 | ≥ 12 | - |
| | Periplasm | 11 ± 2 | 3 ± 1 | 0 | 3 | ≥ 1,087 | - |
| | Spheroplast | 7 ± 0 | 0 ± 0 | 0 | 17 | ≥ 687 | - |
| | Secreted | 11 ± 2 | 3 ± 1 | 0 | 3 | ≥ 1,099 | 62 |
| | Total | 17 ± 2 | 3 ± 1 | 0 | 5 | ≥ 1,786 | - |
| scFvT | Media | 0 ± 0 | 0 ± 0 | 0 | 20 | ≥ 29 | - |
| | Periplasm | 6 ± 1 | 1 ± 0 | 0 | 4 | ≥ 574 | - |
| | Spheroplast | 5 ± 1 | 1 ± 0 | 0 | 48 | ≥ 520 | - |
| | Secreted | 6 ± 1 | 1 ± 0 | 0 | 4 | ≥ 603 | 54 |
| | Total | 11 ± 0 | 1 ± 0 | 0 | 7 | ≥ 1,123 | - |
| DsbA E1 | mg/L | Max (IP) | 20 IP | Pre-induction | Max IP/20 IP | IP/UI | Secretion (%) |
| --- | --- | --- | --- | --- | --- | --- | --- |
| scFvβ | Media | 4 ± 2 | 0 ± 0 | 0 | 10 | ≥ 385 | - |
| | Periplasm | 348 ± 61 | 125 ± 29 | 0 | 3 | ≥ 34,848 | - |
| | Spheroplast | 220 ± 2 | 15 ± 1 | 0 | 15 | ≥ 22,016 | - |
| | Secreted | 352 ± 73 | 125 ± 35 | 0 | 3 | ≥ 35,234 | 62 |
| | Total | 572 ± 72 | 140 ± 36 | 0 | 4 | ≥ 57,250 | - |
| scFvT | Media | 10 ± 2 | 1 ± 1 | 0 | 18 | ≥ 998 | - |
| | Periplasm | 200 ± 33 | 60 ± 9 | 0 | 3 | ≥ 20,023 | - |
| | Spheroplast | 181 ± 25 | 4 ± 3 | 0 | 42 | ≥ 18,132 | - |
| | Secreted | 210 ± 34 | 60 ± 10 | 0 | 3 | ≥ 21,020 | 54 |
| | Total | 392 ± 10 | 64 ± 8 | 0 | 6 | ≥ 39,152 | - |
7

## Slide 8
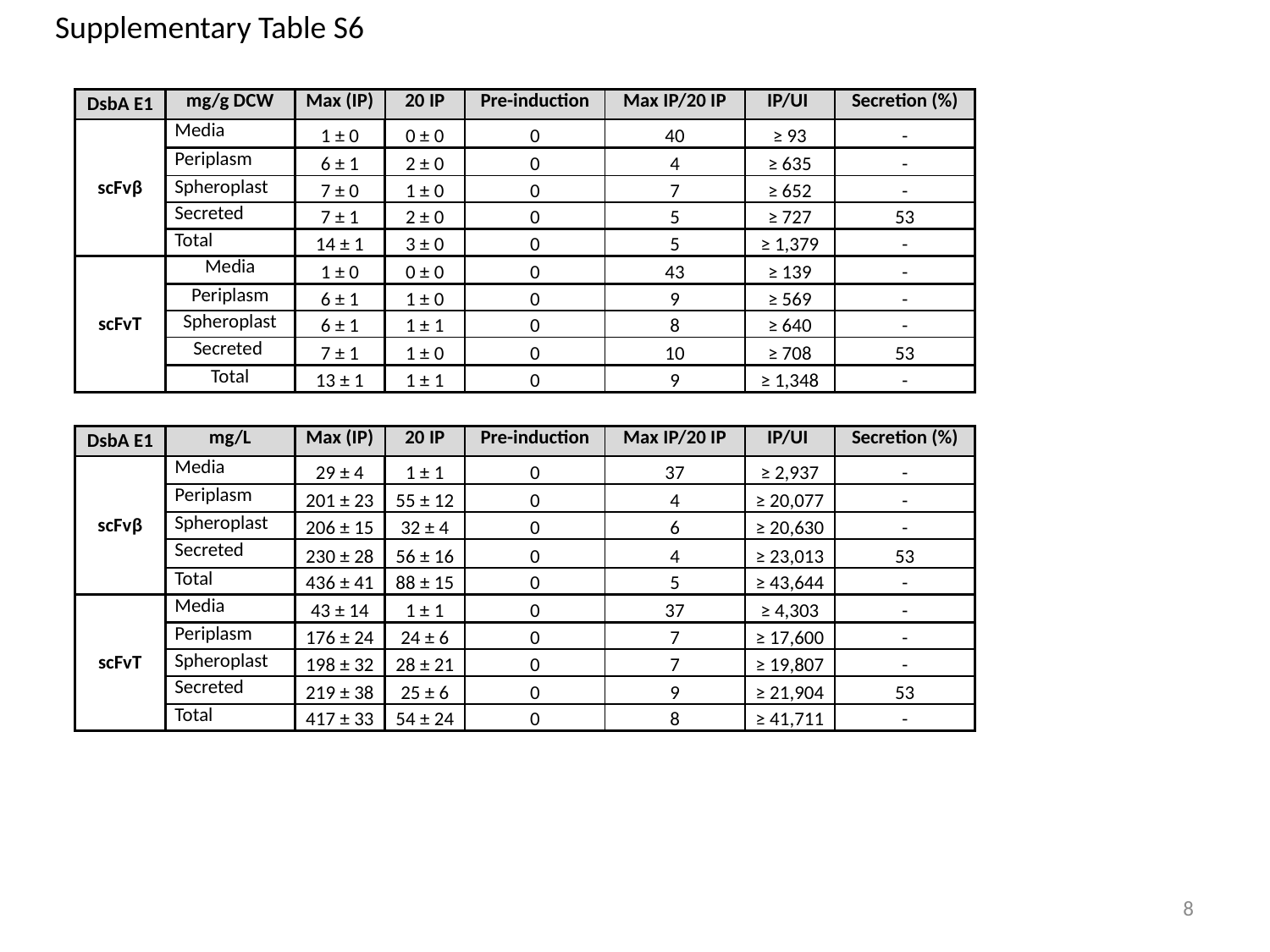

Supplementary Table S6
| DsbA E1 | mg/g DCW | Max (IP) | 20 IP | Pre-induction | Max IP/20 IP | IP/UI | Secretion (%) |
| --- | --- | --- | --- | --- | --- | --- | --- |
| scFvβ | Media | 1 ± 0 | 0 ± 0 | 0 | 40 | ≥ 93 | - |
| | Periplasm | 6 ± 1 | 2 ± 0 | 0 | 4 | ≥ 635 | - |
| | Spheroplast | 7 ± 0 | 1 ± 0 | 0 | 7 | ≥ 652 | - |
| | Secreted | 7 ± 1 | 2 ± 0 | 0 | 5 | ≥ 727 | 53 |
| | Total | 14 ± 1 | 3 ± 0 | 0 | 5 | ≥ 1,379 | - |
| scFvT | Media | 1 ± 0 | 0 ± 0 | 0 | 43 | ≥ 139 | - |
| | Periplasm | 6 ± 1 | 1 ± 0 | 0 | 9 | ≥ 569 | - |
| | Spheroplast | 6 ± 1 | 1 ± 1 | 0 | 8 | ≥ 640 | - |
| | Secreted | 7 ± 1 | 1 ± 0 | 0 | 10 | ≥ 708 | 53 |
| | Total | 13 ± 1 | 1 ± 1 | 0 | 9 | ≥ 1,348 | - |
| DsbA E1 | mg/L | Max (IP) | 20 IP | Pre-induction | Max IP/20 IP | IP/UI | Secretion (%) |
| --- | --- | --- | --- | --- | --- | --- | --- |
| scFvβ | Media | 29 ± 4 | 1 ± 1 | 0 | 37 | ≥ 2,937 | - |
| | Periplasm | 201 ± 23 | 55 ± 12 | 0 | 4 | ≥ 20,077 | - |
| | Spheroplast | 206 ± 15 | 32 ± 4 | 0 | 6 | ≥ 20,630 | - |
| | Secreted | 230 ± 28 | 56 ± 16 | 0 | 4 | ≥ 23,013 | 53 |
| | Total | 436 ± 41 | 88 ± 15 | 0 | 5 | ≥ 43,644 | - |
| scFvT | Media | 43 ± 14 | 1 ± 1 | 0 | 37 | ≥ 4,303 | - |
| | Periplasm | 176 ± 24 | 24 ± 6 | 0 | 7 | ≥ 17,600 | - |
| | Spheroplast | 198 ± 32 | 28 ± 21 | 0 | 7 | ≥ 19,807 | - |
| | Secreted | 219 ± 38 | 25 ± 6 | 0 | 9 | ≥ 21,904 | 53 |
| | Total | 417 ± 33 | 54 ± 24 | 0 | 8 | ≥ 41,711 | - |
8

## Slide 9
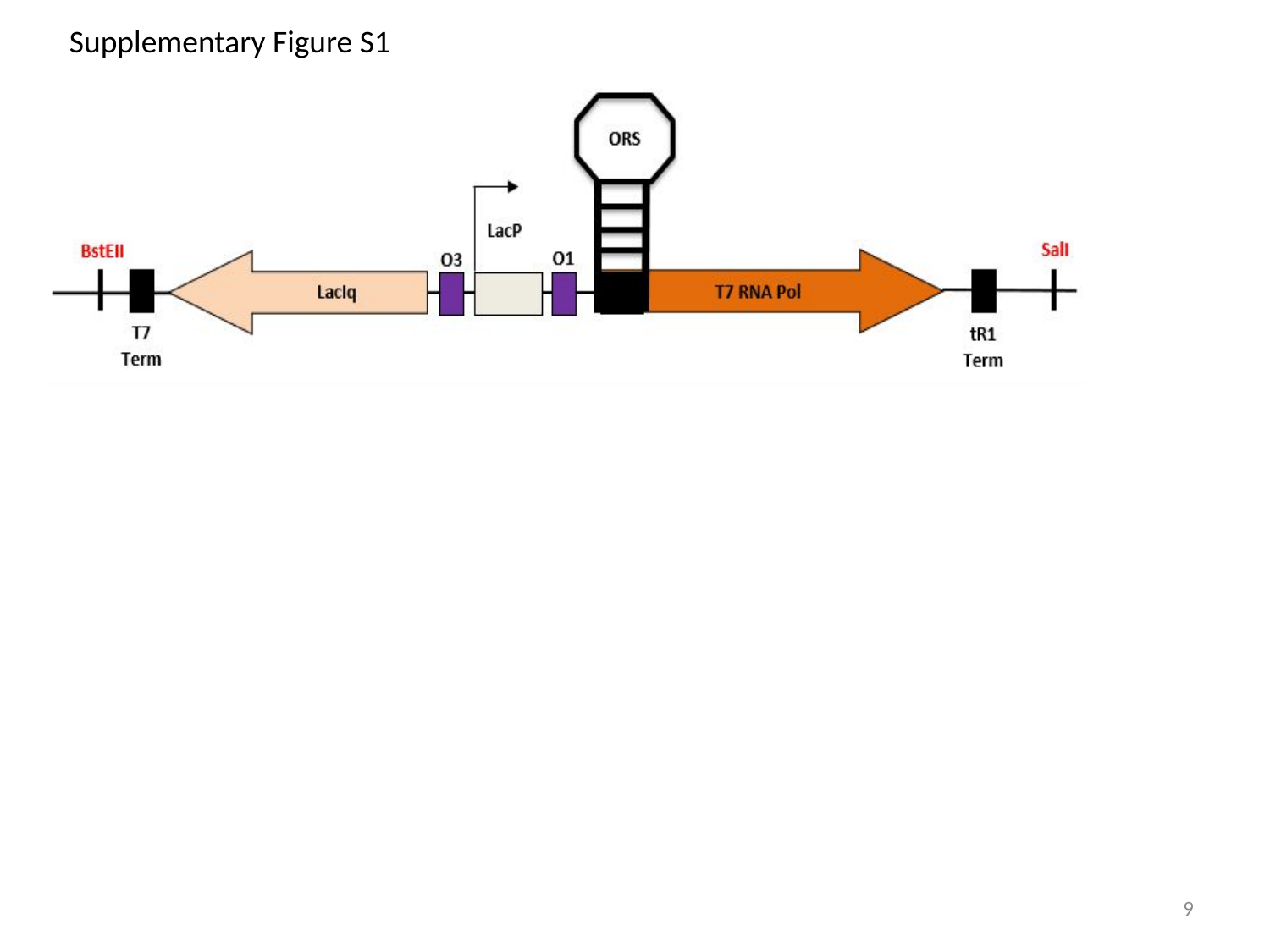

Supplementary Figure S1
9

## Slide 10
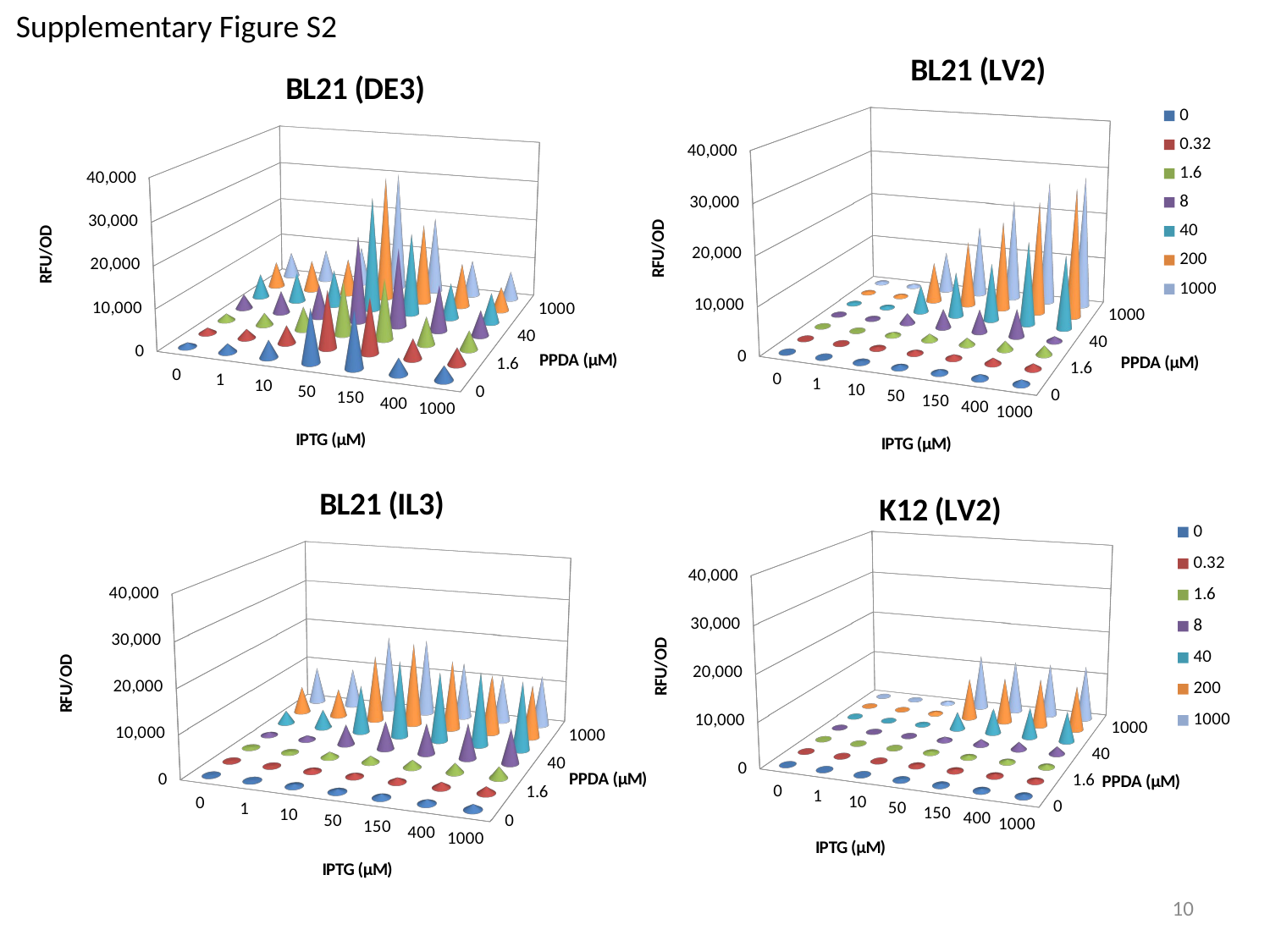

Supplementary Figure S2
[unsupported chart]
[unsupported chart]
[unsupported chart]
[unsupported chart]
10

## Slide 11
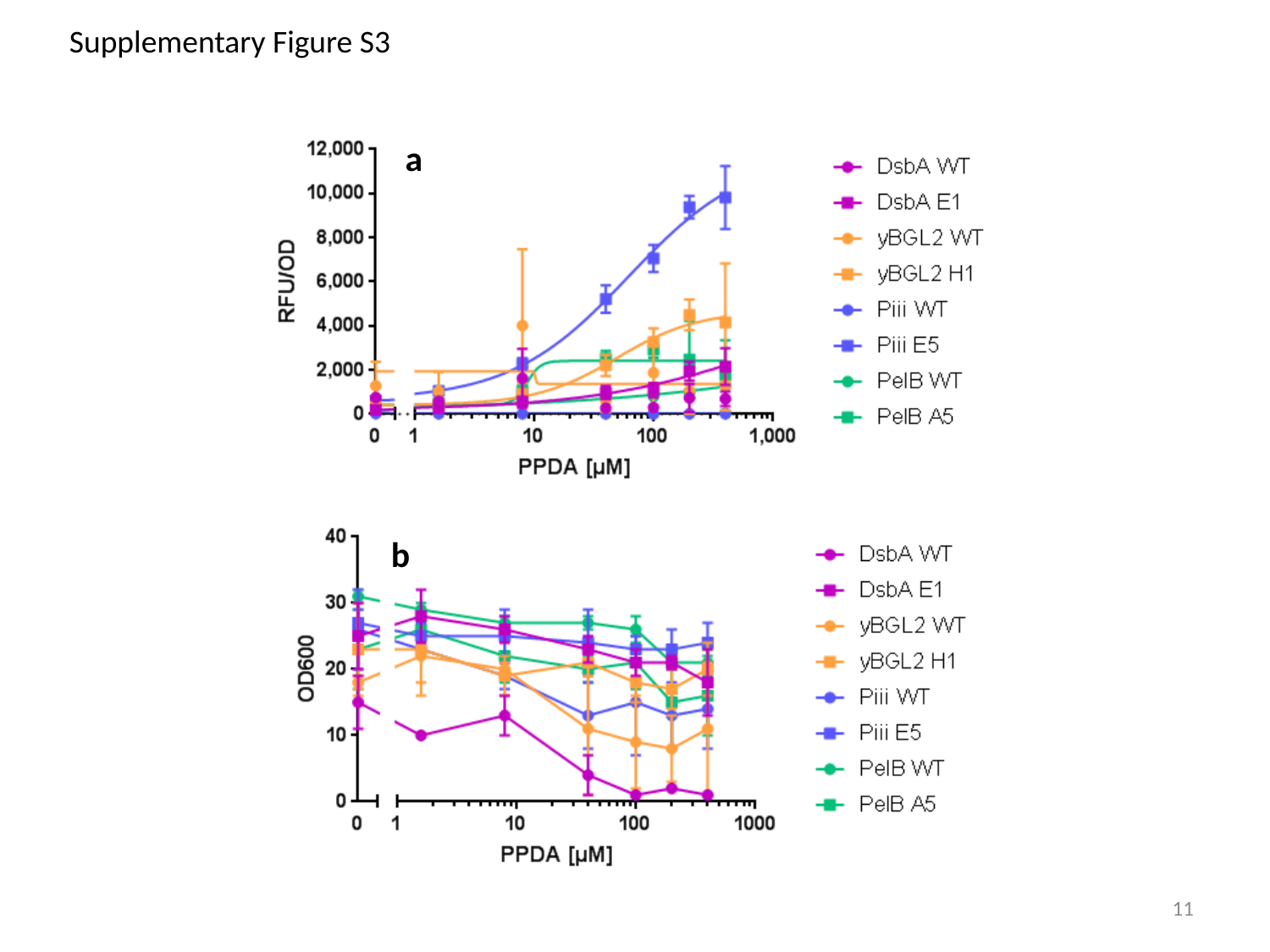

Supplementary Figure S3
a
b
11

## Slide 12
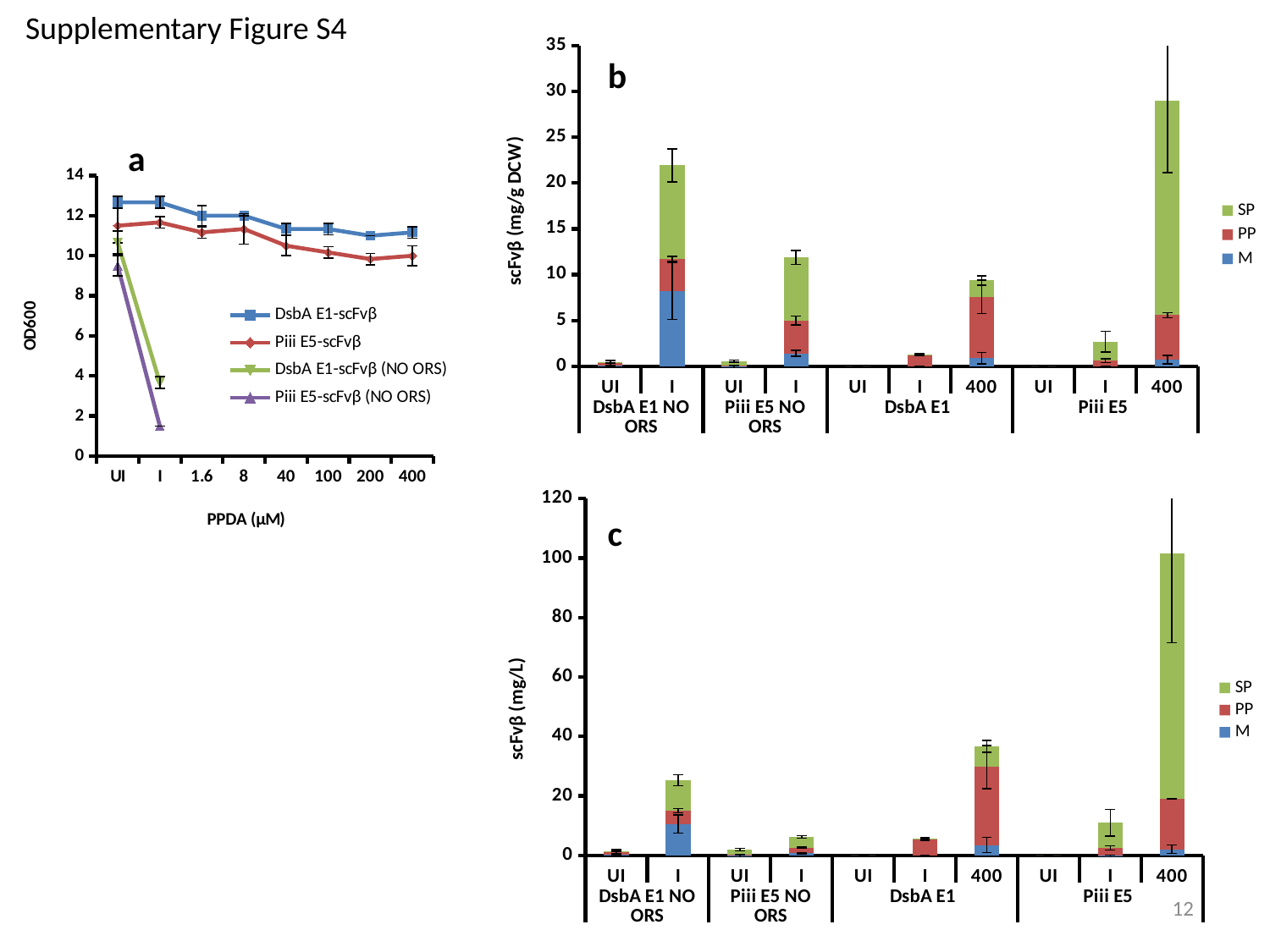

Supplementary Figure S4
### Chart
| Category | M | PP | SP |
|---|---|---|---|
| UI | 0.072874469503422 | 0.296495711489567 | 0.120591702909004 |
| I | 8.205518512524627 | 3.511441446713875 | 10.19558395577544 |
| UI | 0.0105479532136024 | 0.0986519880187584 | 0.462944231542311 |
| I | 1.413135014117129 | 3.580678054811086 | 6.894249077307634 |
| UI | 0.0 | 0.0 | 0.0 |
| I | 0.0 | 1.238728894721006 | 0.0655753192144353 |
| 400 | 0.88444839753282 | 6.697063863750928 | 1.774955635739957 |
| UI | 0.0 | 0.0 | 0.0 |
| I | 0.0 | 0.605937755681525 | 2.086474602002715 |
| 400 | 0.72242012465517 | 4.85346268542102 | 23.39375844806129 |b
a
[unsupported chart]
### Chart
| Category | M | PP | SP |
|---|---|---|---|
| UI | 0.273731709744849 | 1.086703991988712 | 0.120591702909004 |
| I | 10.57765685656461 | 4.524102143930079 | 10.19558395577544 |
| UI | 0.0368957165134875 | 0.32774474804985 | 1.541583433792213 |
| I | 0.741895882411495 | 1.87985597877582 | 3.619480765586508 |
| UI | 0.0 | 0.0 | 0.0 |
| I | 0.0 | 5.49446765525271 | 0.290931366480271 |
| 400 | 3.499217708830358 | 26.22338043202521 | 6.934937296260705 |
| UI | 0.0 | 0.0 | 0.0 |
| I | 0.0354518499419879 | 2.470439063891406 | 8.505721524622976 |
| 400 | 2.092816397790344 | 16.95294496457397 | 82.60778830547117 |c
12

## Slide 13
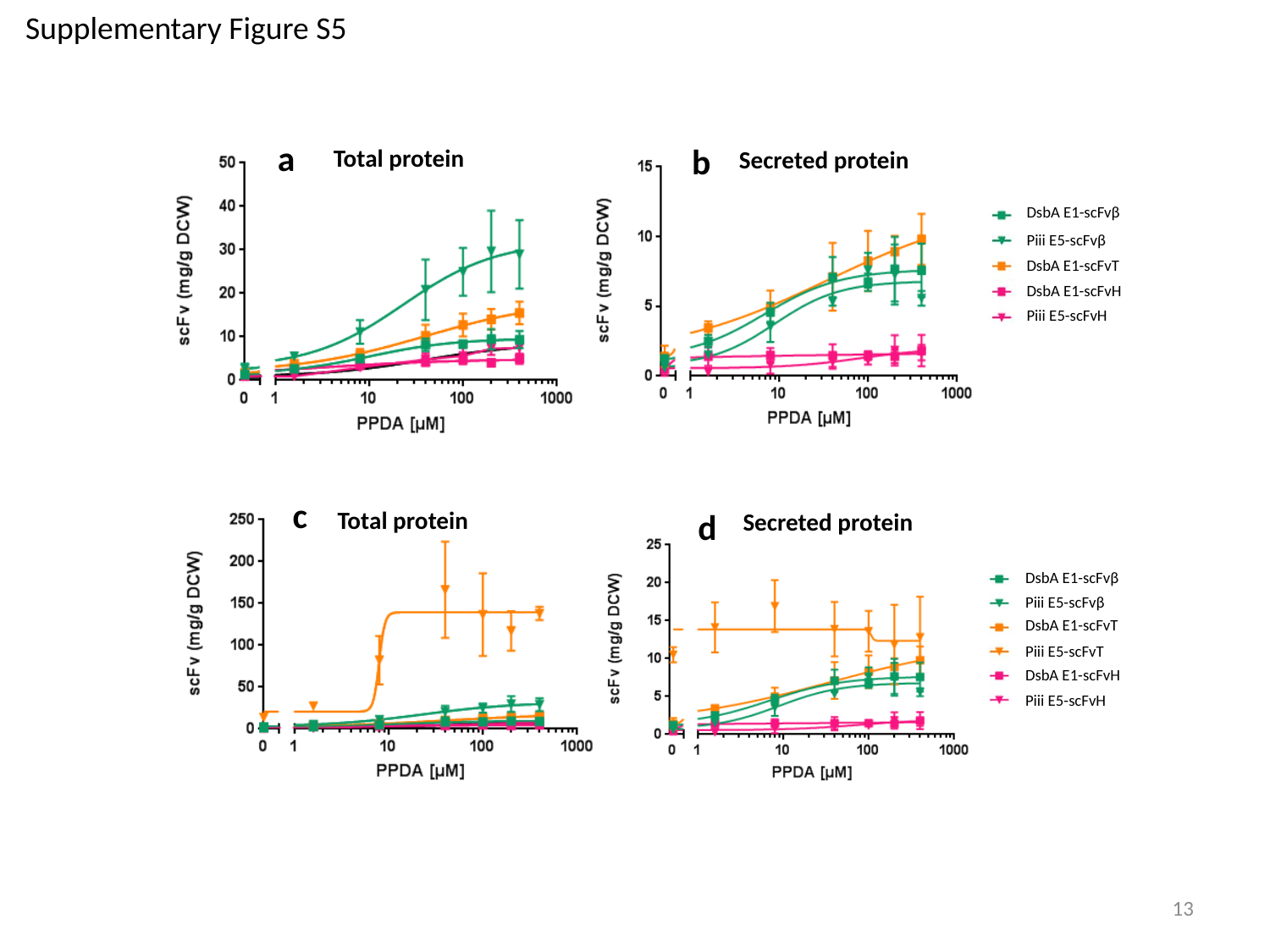

Supplementary Figure S5
a
b
Total protein
Secreted protein
DsbA E1-scFvβ
Piii E5-scFvβ
DsbA E1-scFvT
DsbA E1-scFvH
Piii E5-scFvH
c
d
Total protein
Secreted protein
DsbA E1-scFvβ
Piii E5-scFvβ
DsbA E1-scFvT
Piii E5-scFvT
DsbA E1-scFvH
Piii E5-scFvH
13

## Slide 14
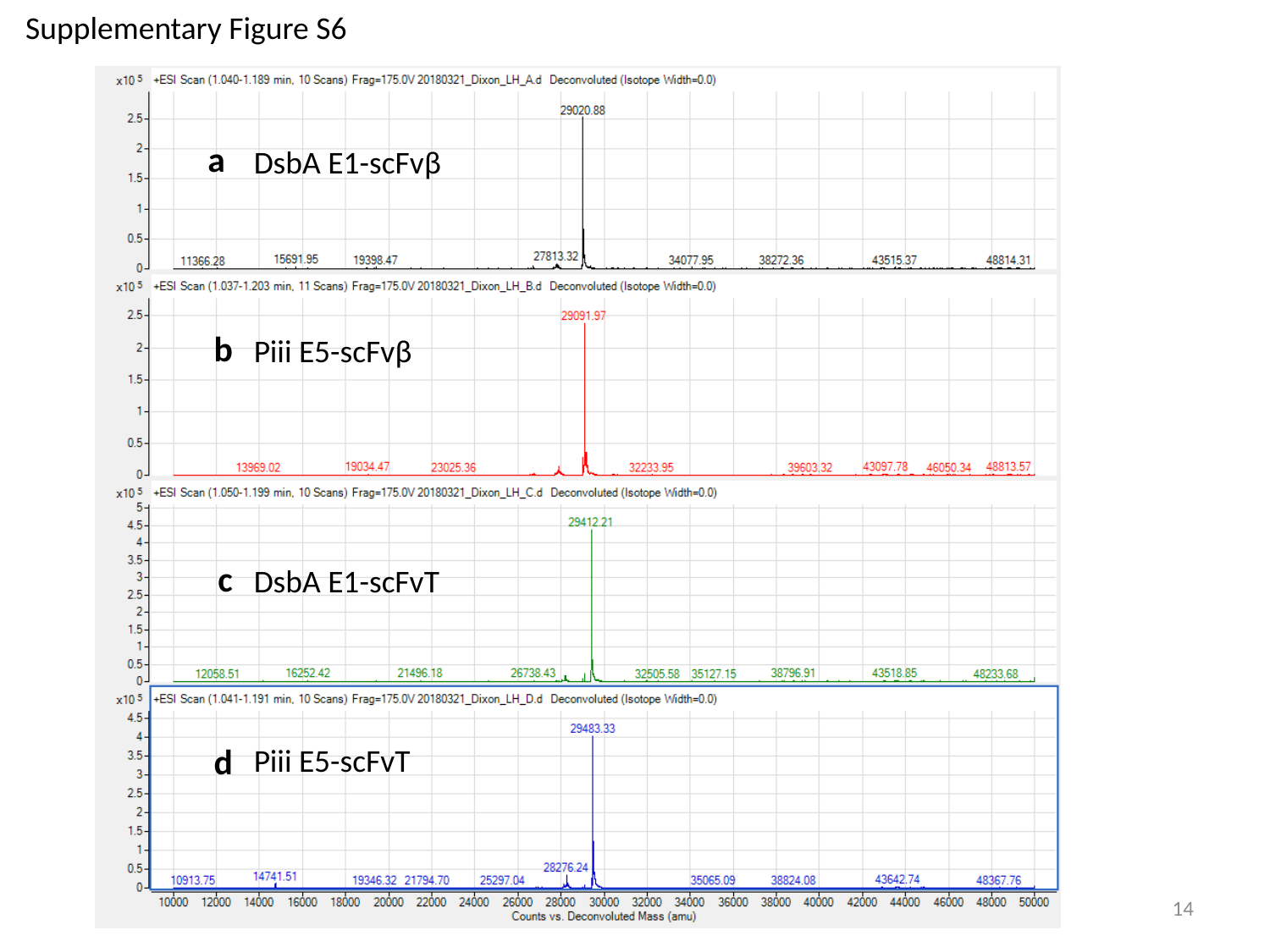

Supplementary Figure S6
a
b
c
d
DsbA E1-scFvβ
Piii E5-scFvβ
DsbA E1-scFvT
Piii E5-scFvT
14

## Slide 15
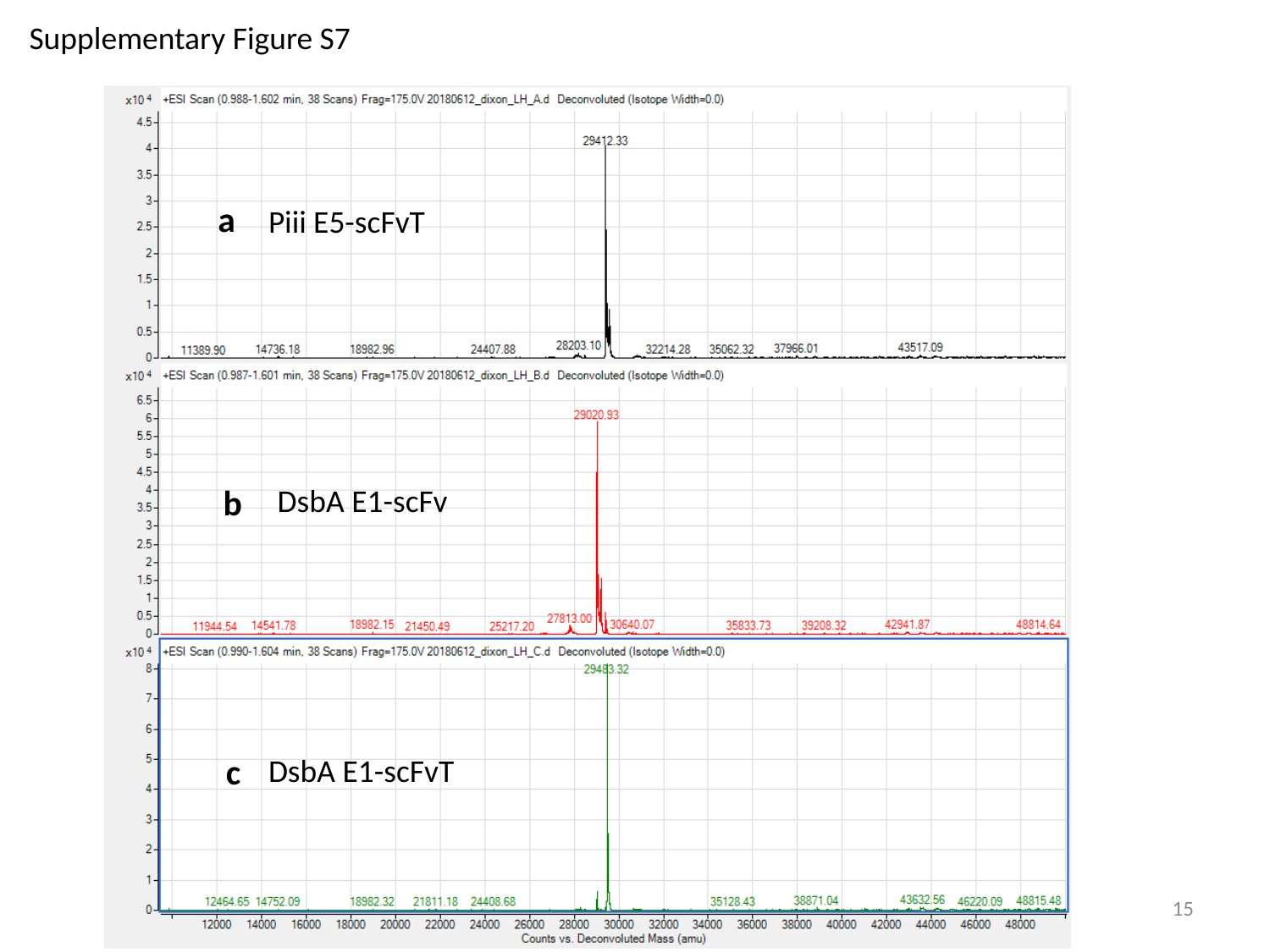

Supplementary Figure S7
a
b
c
Piii E5-scFvT
DsbA E1-scFvT
15

## Slide 16
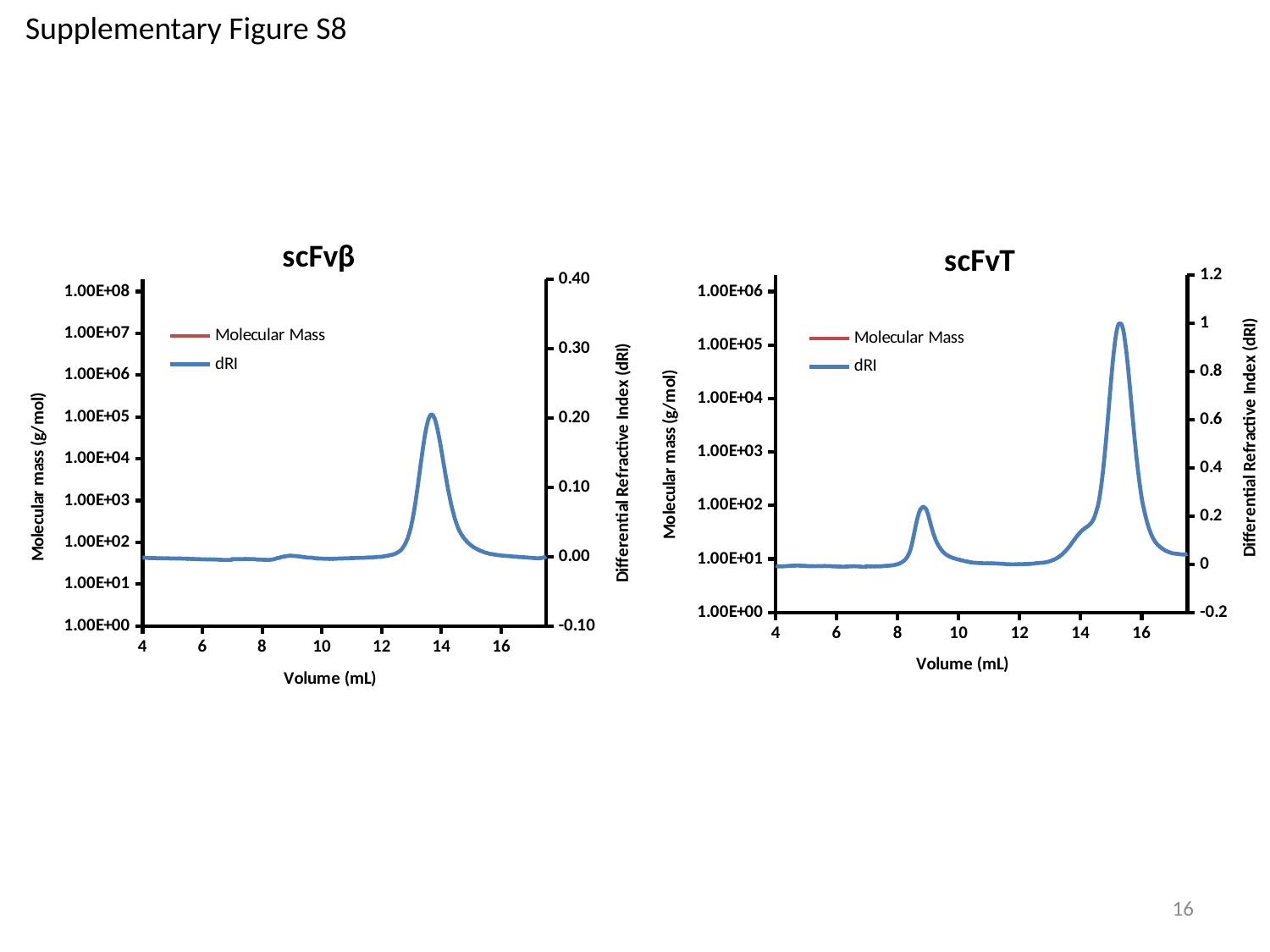

Supplementary Figure S8
### Chart: scFvβ
| Category | | |
|---|---|---|
### Chart: scFvT
| Category | | |
|---|---|---|16

## Slide 17
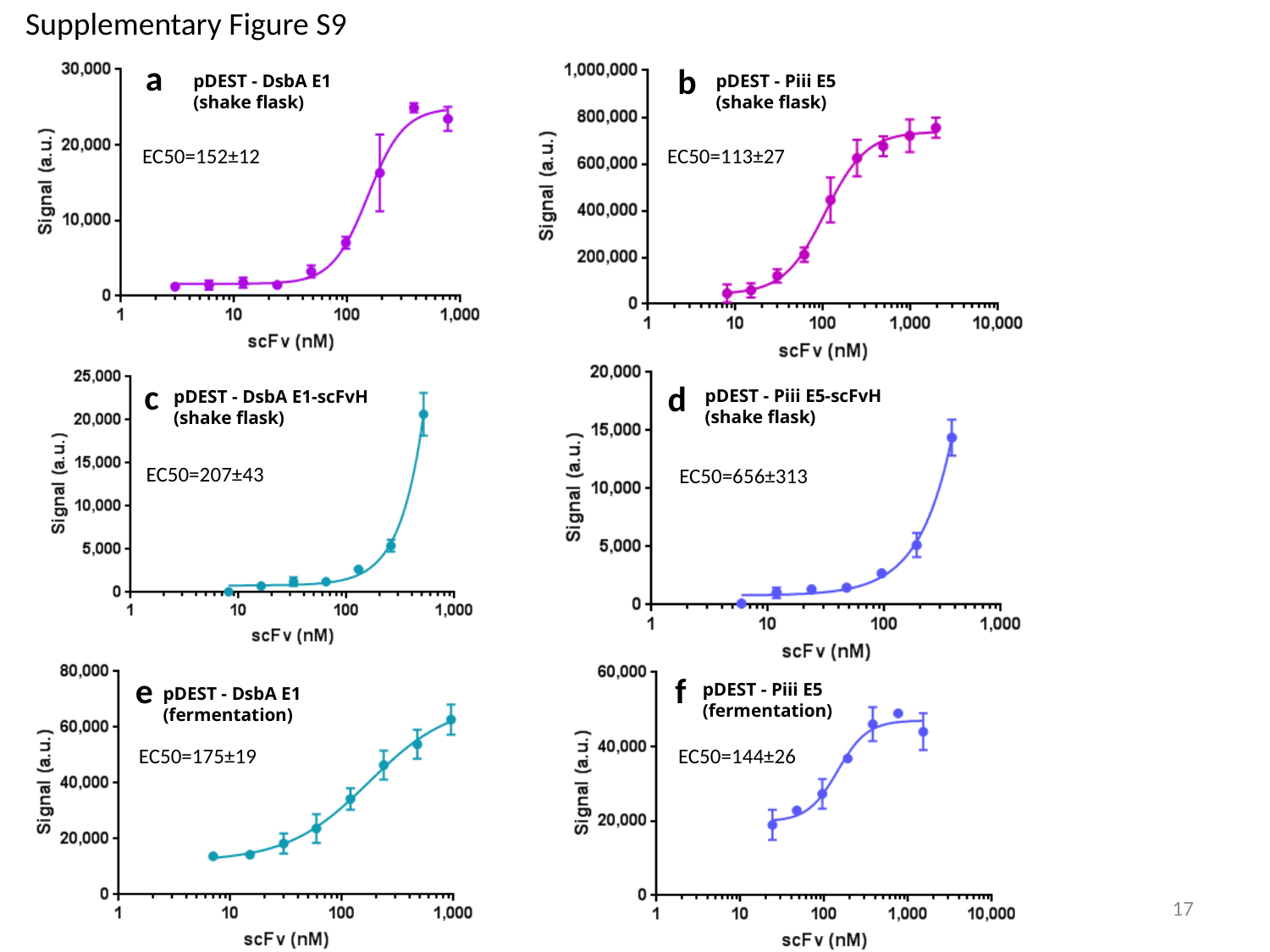

Supplementary Figure S9
a
b
pDEST - Piii E5
(shake flask)
pDEST - DsbA E1
(shake flask)
EC50=152±12
EC50=113±27
c
d
pDEST - Piii E5-scFvH
(shake flask)
pDEST - DsbA E1-scFvH
(shake flask)
EC50=207±43
EC50=656±313
e
f
pDEST - Piii E5
(fermentation)
pDEST - DsbA E1
(fermentation)
EC50=144±26
EC50=175±19
17

## Slide 18
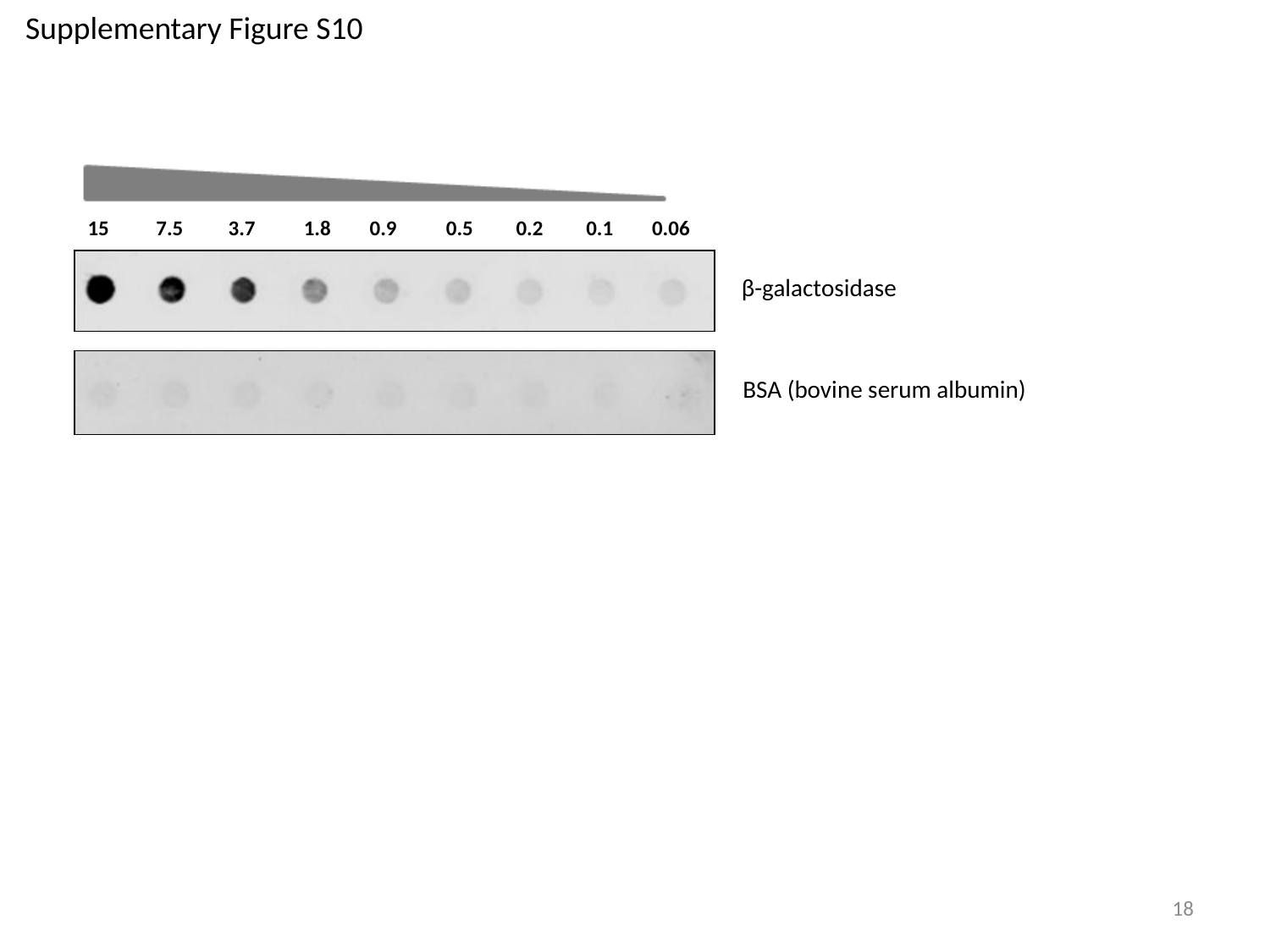

Supplementary Figure S10
15
7.5
3.7
1.8
0.9
0.5
0.2
0.1
0.06
β-galactosidase
ΒSA (bovine serum albumin)
18

## Slide 19
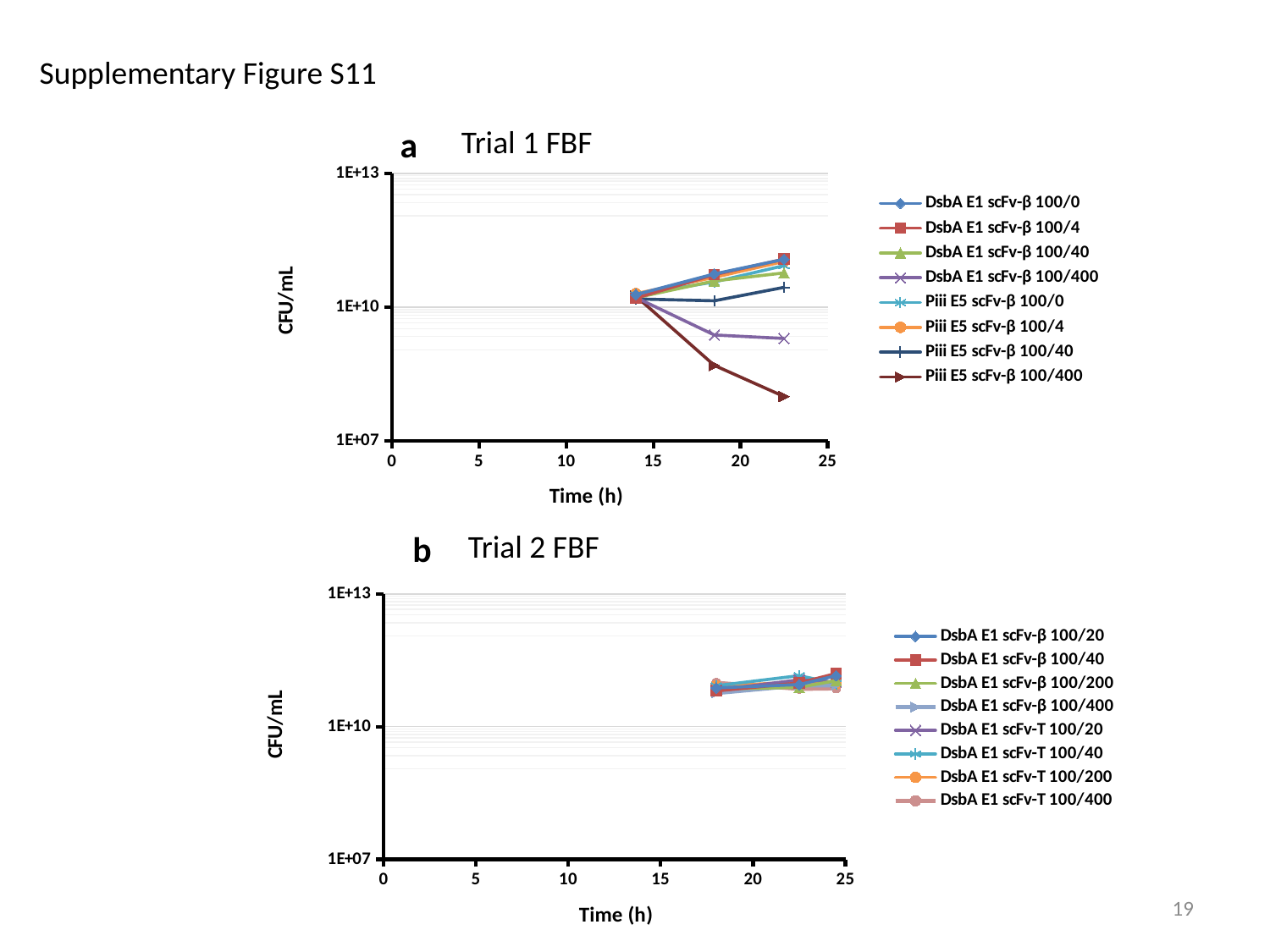

Supplementary Figure S11
Trial 1 FBF
a
### Chart
| Category | DsbA E1 scFv-β 100/0 | DsbA E1 scFv-β 100/4 | DsbA E1 scFv-β 100/40 | DsbA E1 scFv-β 100/400 | Piii E5 scFv-β 100/0 | Piii E5 scFv-β 100/4 | Piii E5 scFv-β 100/40 | Piii E5 scFv-β 100/400 |
|---|---|---|---|---|---|---|---|---|Trial 2 FBF
b
### Chart
| Category | DsbA E1 scFv-β 100/20 | DsbA E1 scFv-β 100/40 | DsbA E1 scFv-β 100/200 | DsbA E1 scFv-β 100/400 | DsbA E1 scFv-T 100/20 | DsbA E1 scFv-T 100/40 | DsbA E1 scFv-T 100/200 | DsbA E1 scFv-T 100/400 |
|---|---|---|---|---|---|---|---|---|19

## Slide 20
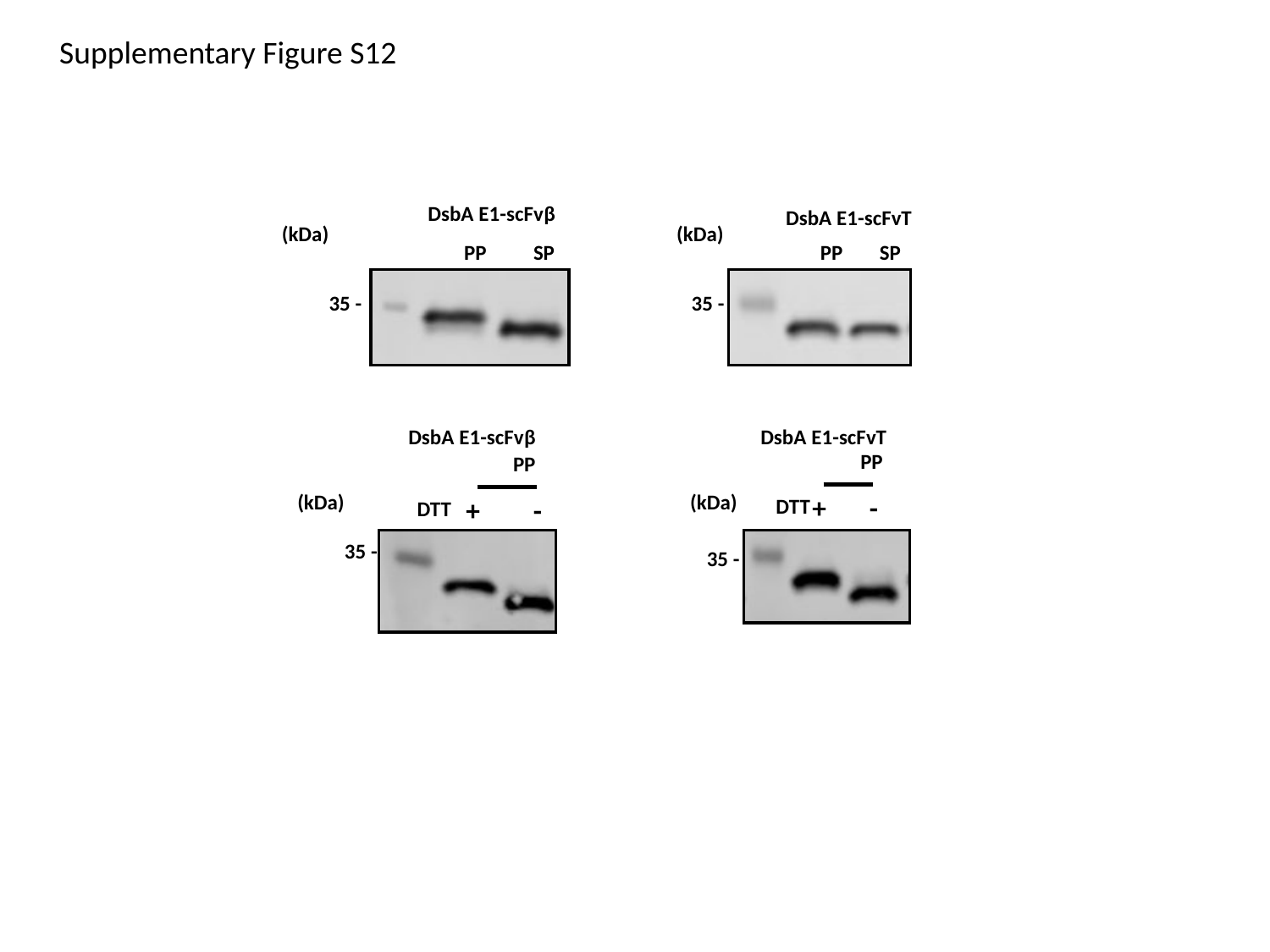

Supplementary Figure S12
DsbA E1-scFvβ
DsbA E1-scFvT
(kDa)
(kDa)
PP
SP
PP
SP
35 -
35 -
DsbA E1-scFvβ
DsbA E1-scFvT
PP
-
+
DTT
PP
-
+
DTT
(kDa)
(kDa)
35 -
35 -

## Slide 21
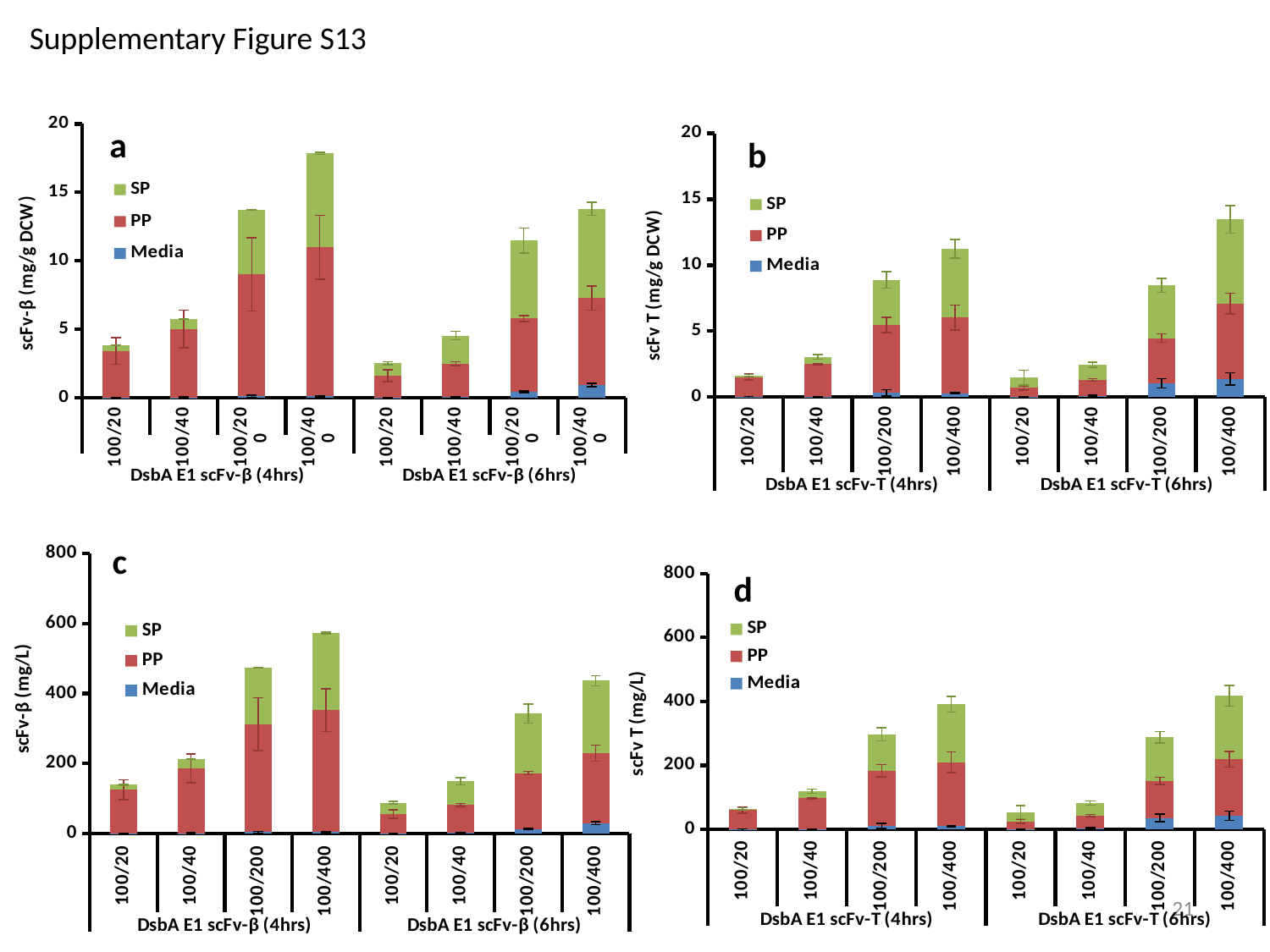

Supplementary Figure S13
### Chart
| Category | Media | PP | SP |
|---|---|---|---|
| 100/20 | 0.0107771686146817 | 3.42116962866198 | 0.40721902338195 |
| 100/40 | 0.0291138240375163 | 4.998902661190963 | 0.725245913998342 |
| 100/200 | 0.108798720902894 | 8.913500439009654 | 4.700629190726663 |
| 100/400 | 0.120239171507747 | 10.86975767120564 | 6.867073477007127 |
| 100/20 | 0.023061743907822 | 1.583084121211886 | 0.929472817707815 |
| 100/40 | 0.0664305735996261 | 2.42692293769353 | 2.048014547663506 |
| 100/200 | 0.437529353036147 | 5.351130589220277 | 5.699546913069575 |
| 100/400 | 0.928148789263885 | 6.34531341985111 | 6.520323230422525 |a
### Chart
| Category | Media | PP | SP |
|---|---|---|---|
| 100/20 | 0.0140042740160057 | 1.50920236118435 | 0.109480684720041 |
| 100/40 | 0.0328335762519435 | 2.463270210934761 | 0.544592577567572 |
| 100/200 | 0.330248887730553 | 5.145905777563506 | 3.397551088362216 |
| 100/400 | 0.286172930718058 | 5.743766523345227 | 5.201263131022675 |
| 100/20 | 0.0322838495423912 | 0.665724502594251 | 0.77541161570655 |
| 100/40 | 0.106762749948183 | 1.193435308204694 | 1.145700775757568 |
| 100/200 | 1.043927438409782 | 3.421575698719627 | 4.005293499371593 |
| 100/400 | 1.390849648261151 | 5.688541395624798 | 6.401877300709391 |b
c
### Chart
| Category | Media | PP | SP |
|---|---|---|---|
| 100/20 | 0.392288937574416 | 124.530574483296 | 14.822772451103 |
| 100/40 | 1.076046936426603 | 184.7594423576181 | 26.80508898137871 |
| 100/200 | 3.762259768822088 | 308.2288451809538 | 162.5477574153278 |
| 100/400 | 3.85486783853836 | 348.4844309388524 | 220.1583756728485 |
| 100/20 | 0.800703748479581 | 54.96468068847662 | 32.27129623081534 |
| 100/40 | 2.176265591123746 | 79.5059954388401 | 67.09295658145636 |
| 100/200 | 13.0471253075379 | 159.5707141705486 | 169.960488947735 |
| 100/400 | 29.36662769230929 | 200.7657166040891 | 206.3030270105687 |
### Chart
| Category | Media | PP | SP |
|---|---|---|---|
| 100/20 | 0.552888738151905 | 59.58330921955813 | 4.322297432747223 |
| 100/40 | 1.28937453941382 | 96.73262118340806 | 21.38615052107857 |
| 100/200 | 11.0501277834643 | 172.1820073172753 | 113.6820594165995 |
| 100/400 | 9.975988364831514 | 200.2277010038145 | 181.3160327474502 |
| 100/20 | 1.175132123343042 | 24.23237189443073 | 28.22498281171842 |
| 100/40 | 3.602175183251694 | 40.26650729882641 | 38.65594417406032 |
| 100/200 | 35.5144114547007 | 116.4020052704417 | 136.2600848486216 |
| 100/400 | 43.0328881172 | 176.0034707806312 | 198.0740836839481 |d
21
